# Structural basis of *Fusarium* myosin I inhibition by phenamacril

**DOI:** 10.1371/journal.ppat.1008323

**Published:** 2020-03-12

**Authors:** Yuxin Zhou, X. Edward Zhou, Yuanping Gong, Yuanye Zhu, Xiaoman Cao, Joseph S. Brunzelle, H. Eric Xu, Mingguo Zhou, Karsten Melcher, Feng Zhang

**Affiliations:** 1 Key Laboratory of Pesticide, College of Plant Protection, Nanjing Agricultural University, Nanjing, China; 2 Center of Cancer and Cell Biology, Program for Structural Biology, Van Andel Institute, Grand Rapids, Michigan, United States of America; 3 Northwestern University Synchrotron Research Center, Life Sciences Collaborative Access Team, Northwestern University, Argonne, Illinois, United States of America; 4 Center for Structure and Function of Drug Targets, The CAS-Key Laboratory of Receptor Research, Shanghai Institute of Materia Medica, Chinese Academy of Sciences, Shanghai, China; United States Department of Agriculture, UNITED STATES

## Abstract

*Fusarium* is a genus of filamentous fungi that includes species that cause devastating diseases in major staple crops, such as wheat, maize, rice, and barley, resulting in severe yield losses and mycotoxin contamination of infected grains. Phenamacril is a novel fungicide that is considered environmentally benign due to its exceptional specificity; it inhibits the ATPase activity of the sole class I myosin of only a subset of *Fusarium* species including the major plant pathogens *F*. *graminearum*, *F*. *asiaticum and F*. *fujikuroi*. To understand the underlying mechanisms of inhibition, species specificity, and resistance mutations, we have determined the crystal structure of phenamacril-bound *F*. *graminearum* myosin I. Phenamacril binds in the actin-binding cleft in a new allosteric pocket that contains the central residue of the regulatory Switch 2 loop and that is collapsed in the structure of a myosin with closed actin-binding cleft, suggesting that pocket occupancy blocks cleft closure. We have further identified a single, transferable phenamacril-binding residue found exclusively in phenamacril-sensitive myosins to confer phenamacril selectivity.

## Author summary

Phenamacril is a recently identified myosin I inhibitor that is a potent and highly species-specific and myosin subtype-selective fungicide. We report the high-resolution structure of the phenamacril-bound myosin I motor domain of the major crop pathogen *Fusarium graminearum*, providing insight into the molecular mechanism of phenamacril action and resistance. These results are of broad significance for understanding the mode of actions of myosin-based fungicides and for designing novel myosin I inhibitors for crop protection and for treatment of human myosin dysfunction diseases.

## Introduction

*Fusarium graminearum* and *F*. *asiaticum* are plant pathogens that cause head blight, root rot, and seedling blight, diseases in wheat, maize, and barley, while *F*. *fujikuroi* is the causal agent of rice bakanae disease [1, 2]. These pathogens cause both major yield losses and contamination of infested grains with mycotoxins, including deoxynivalenol, that are toxic to humans and animals. Phenamacril (experimental code JS399-19) is an effective and highly species-specific fungicide, even though it targets the conserved motor domain of myosins, which are found in all eukaryotes [3, 4]. How phenamacril achieves its specificity is unknown.

**Fig 1:**
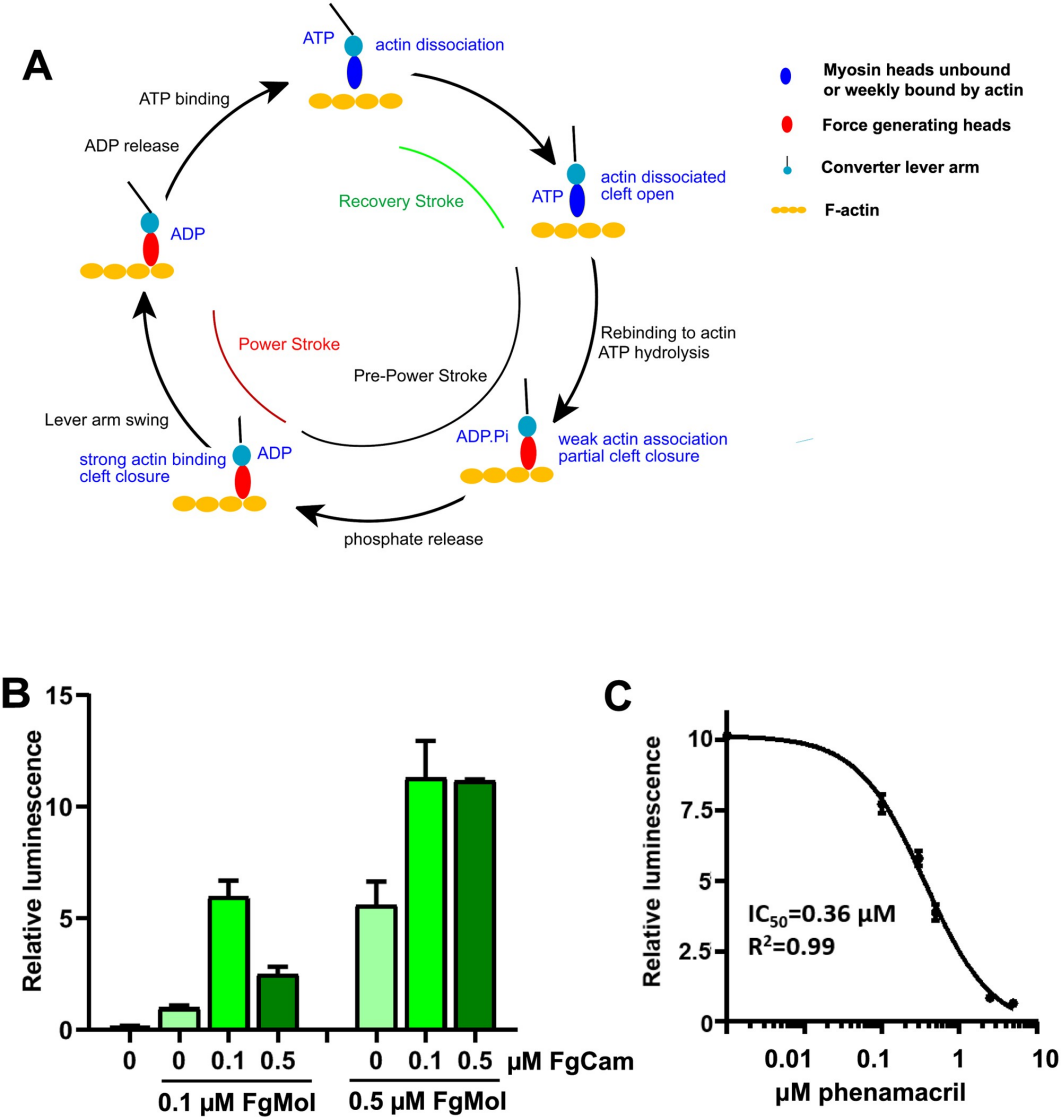
FgMyoI ATPase activity. (A) Myosin/actin catalytic cycle (modified from [13]). ATP-bound myosin is dissociated from actin and becomes rapidly hydrolyzed in the catalytic site. Release of the hydrolyzed phosphate is slow and requires actin binding for acceleration. Initial weak electrostatic actin binding partially closes the actin-binding cleft and stabilizes a switch loop movement. This allows concerted phosphate release, full cleft closure, actin binding and lever arm movement (power stroke). This is followed by ADP release, which allows rapid ATP rebinding, actin dissociation, and a lever arm recovery stroke. (B) Calmodulin dependence of the FgMyoI activity measured by ATP-Glo ATPase luminescence assay. (C) Phenamacril inhibits the ATPase activity of FgMyoI; n = 3, error bars = SD.

Myosins comprise a superfamily of ATP-driven molecular motors involved in several cellular processes, including muscle contraction, vesicle transport, cytokinesis, organelle movement, and sensory transduction. Based on sequence homology, they are grouped into 35 classes, of which the myosins II form the “conventional” myosins responsible for muscle contraction (for review see [5, 6]). Myosins have a highly conserved ATP- and myosin-binding motor domain, a force transducing lever arm containing one to several Ile/Gln (IQ) motifs, and a variable cargo interacting tail domain. The IQ motifs function as binding domain for calmodulin and calmodulin-like myosin light chains, which provide rigidity to the lever arm and increase motor activity [7, 8]. ATP hydrolysis and release of the hydrolysis products is coupled to actin binding and a large “powerstroke” lever arm movement on actin (Fig 1A) [9–13].

Numerous mutations that cause gain or loss of myosin motor function have been implicated in human diseases and genetic syndromes, including cardiomyopathies, cancer progression and metastasis, deafness, and neurological disorders [6, 14–17]. Selective myosin inhibitors and activators are therefore both important tools for the study of myosin function and have therapeutic potential for the treatment of myosin dysfunction or overactivity. At present, there are only few myosin inhibitors including the myosin I inhibitor pentachloropseudilin (PCIP) [18], the myosin II inhibitors blebbistatin [19], N-benzyl-p-toluene sulphonamide (BTS) [20], and 2,3-Butanedione monoxime (BDM) [21], the beta-cardiac myosin inhibitor mavacamten [22, 23], the myosin V inhibitors pentabromopseudilin (PBP) [24] and MyoVin-1 [25], and the myosin VI inhibitor 2,4,6-triiodophenol (TIP) [26]. These compounds have been developed to inhibit mammalian myosins. In contrast, phenamacril is the only reported inhibitor that is specific for some plant pathogens [4, 27, 28].

Phenamacril is a novel reversible and non-competitive inhibitor of *Fusarium* myosin I[27]. All *Fusarium* species have only a single class I myosin. Phenamacril functions as a potent fungicide against selective *Fusarium* spp. such as *F*. *graminearum*, *F*. *asiaticum*, and *F*. *fujikuroi* [27, 29], as well as human pathogenic strains of *F*. *oxysporum* [30], but has low or no activity against other *Fusarium* spp., other fungi, myosins 1B, 1E, or 2 from *Dictyostelium* or mammalian Myo1c [27, 28]. Phenamacril shows excellent activity in the field and has been extensively used to control *Fusarium* head blight (FHB) and rice bakanae disease in China [30–32]. In addition to its high activity, phenamacril has low toxicity and is environment-friendly due to its selectivity [27]. However, its use is limited by the development of resistance due to several point mutations in the myosin motor domain (Table 1) [4, 28, 30, 32, 33]. To identify the phenamacril binding site and to elucidate the structural basis of its mechanism of action, we have determined the crystal structure of phenamacril bound to the ATPγS-complex of the motor domain and IQ1 [FgMyoI(1–736)] of the *F*. *graminearum* myosin I encoded by the *myo5* gene. The structure shows that phenamacril binds in a pocket inside of the actin-binding cleft and suggests the structural basis of phenamacril action and resistance mechanisms.

**Table 1:** List of amino-acid (AA) changes in phenamacril resistance mutations in different *Fusarium* species.

AA position and change	Resistance level	Test method	Species
A135→T	LR ^a^	Mycelial growth-inhibitory effect on phenamacril-amended agar	*Fusarium asiaticum* [33]
V151→M			
P204→S			
I434→M			
A577→T			
R580→G/H			
I581→F			
S418→R	MR ^b^		
I424→R			
A577→G			
K216→R/E	HR ^c^		
S217→P/L			
E420→K/G/D			
S217→L/P	HR	Inhibition of the actin-activated ATPase activity/ Mycelial growth-inhibitory effect on phenamacril-amended agar	*F*. *graminearum* [4, 28]
E420→K		Mycelial growth-inhibitory effect on phenamacril-amended agar	
K216→E		Mycelial growth-inhibitory effect on phenamacril-amended agar	
S418→R			
S219→L/P	HR		*F*. *fujikuroi* [32]
V151→A	LR/MR		*F*. *oxysporum* [30]
S418→T			

^a^ LR: low resistance;

^b^ MR: intermediate resistance;

^c^ HR: high resistance.

## Results

### The ATPase activity of FgMyoI(1–736) is modulated by calmodulin and inhibited by phenamacril

We expressed the N-terminal 736 amino acids of *F*. *graminearum* myosin I (FgMyoI) comprising the motor domain and the first IQ motif of the lever arm (Fig 1B) in Sf9 insect cells and *F*. *graminearum* calmodulin (FgCaM) in *E*. *coli*. As reported previously [27], substochiometric amounts of calmodulin were sufficient for full stimulation of the ATPase activity of FgMyoI(1–736), while a five-fold molar excess of calmodulin over myosin reduced the ATPase activity (Fig 1B). Phenamacril inhibited the ATPase activity of FgMyoI(1–736) with an IC_50_ of 0.36 μM in excellent agreement with the results from a previous study [27] (Fig 1C).

### Phenamacril binds an allosteric pocket in the actin-binding cleft

**Fig 2:**
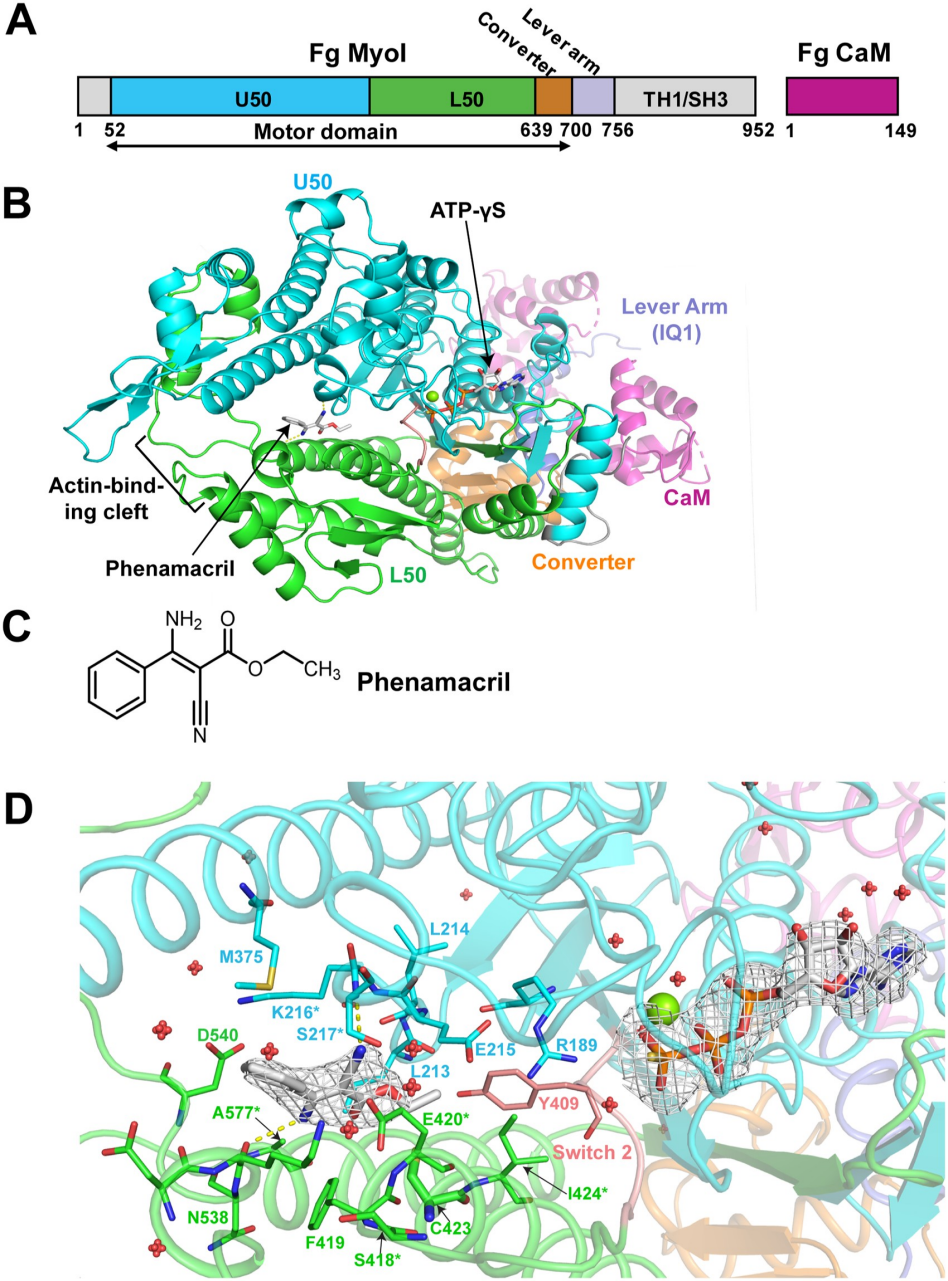
FgMyoI structure. (A) Domain map of *F*. *graminearum* FgMyoI encoded by the *myo5* gene in complex with *F*. *graminearum* calmodulin (CaM). (B) Structure overview. (C) Chemical structure of phenamacril. (D) Catalytic center and phenamacril pocket with ligand Fo-Fc omit map contoured at 3.0 σ. The ligand phenamacril shows strong electron density at 3 σ except the ethyl ester tail that is partially disordered due to its flexibility. Pocket residues are shown in thick line presentation, the Mg^2+^ ion as green sphere, and water molecules as red spheres. *: pocket residues that have been found mutated in highly resistant (K216E/R, S217L/P, E420G/K/D) or moderately resistant (S418R, I424R, A577G) *Fusarium* strains.

Phenamacril is an acrylic ester with a phenyl group, an amino group and a cyano group attached at its ethylene backbone (Fig 2C). To identify its binding site and the mechanism of species-specific myosin I inhibition, we co-expressed FgMyoI(1–736) and FgCaM(1–149) in Sf9 cells and determined the crystal structure of FgMyoI(1–736)/FgCaM in complex with phenamacril and ATP-γS at a resolution of 2.65 Å (PDB ID code: 6UI4) (Fig 2B, S1 Fig and Table 2).

**Table 2:** Data collection and refinement statistics.

Data collection	FgMyoI (1–736)/phenamacril
Space group	P2_1_2_1_2_1_
Cell dimensions	
*a*, *b*, *c* (Å)	59.5, 100.3 165.6
α, β, γ (°)	90, 90, 90
Resolution (Å)	49–2.65 (2.78–2.65) *
*R*_sym_ or *R*_merge_	0.14 (1.5)
*I* /σ*I*	11.3 (1.7)
CC1/2	0.999 (0.81)
Completeness (%)	100.0 (99.9)
Redundancy	13.1 (14.1)
**Refinement**	
Resolution (Å)	43–2.65 (2.71–2.65)*
No. reflections	29507 (1822)
*R*_work_ / *R*_free_	21.1/24.4 (32.7/36.7)
Model composition	
No. non-hydrogen atoms	6456
Protein residues	820
Ligand/ion	3
Water	26
*B*-factors	
Protein	92.3
Ligand/ion	61.8
Water	68.7
R.m.s. deviations	
Bond lengths (Å)	0.004
Bond angles (°)	0.938
Ramachandran plot	
Favored (%)	96.65
Outliers (%)	0
MolProbity score	1.49

*Values in parentheses are for highest-resolution shell.

The model of the crystal structure contains 692 FgMyoI amino acid residues (37–728), which consist of U50, L50, and the converter domains, as well as the lever arm IQ1 segment that binds to FgCaM (Fig 2B and S1 Fig). While the myosin domains shows reasonable electron density, the calmodulin component has relatively weak density, consistent with previous discovery that calmodulin is highly dynamic and can adopt various conformations in myosin structures [9, 34, 35] (S1 Fig). We built 125 amino acid residues out of 149 of the whole FgCaM sequence, with many side chains and a few loop regions missing in the model due to relatively weak density of the calmodulin molecule (S1A and S1B Fig). Interestingly, the FgCaM that binds the IQ1 helix of the myosin is in a conformation that is different from most of the published myosin-bound calmodulin conformations (S1C Fig).

The FgMyoI(1–736) structure shows a canonical myosin fold similar to that of *Dictyostelium* myosin I bound to MgADP-VO_4_ [36]. The structure shows a short N-terminal domain followed by the motor domain and the IQ1 helix of the lever arm. The motor domain consists of the upper (U50) and lower (L50) subdomains, which enclose the actin-binding cleft in open state, and the converter, which functionally connects L50 and U50 to the lever arm. The converter adopts the pre-powerstroke conformation that is characteristic for myosins with open actin binding cleft prior to the release of Pi and ADP. ATP-γS, Mg^2+^, and structured water molecules occupy the canonical nucleotide-binding site in U50, while the outside of the cleft entry between U50 and L50 forms the actin binding site (Fig 2B).

The phenamacril pocket has a size of 238 Å^3^ and is located in the actin-binding cleft of the motor domain close to the predicted position in a previous modeling study [27]. Pockets of a similar size range are also found in pre-powerstroke myosins in the absence of phenamacril (e.g., 174 Å^3^ in PDB 1LKX; 275 Å^3^ in 1W9K), indicating that pocket formation is not induced by phenamacril. The pocket is lined by 17 phenamacril-interacting myosin residues: R189, L213, L214, E215, K216, S217, M375, Y409, F419, E420, C423, I424, D536, K537, N538, D540 and A577. The placement of phenamacril in the pocket is well-defined by electron density (Fig 2D). The ethyl ester tail of phenamacril is deeply inserted into the pocket bottom while its phenyl group is oriented at the opening of the pocket. The interaction of phenamacril with the pocket is overall hydrophobic with its carbon backbone associated with hydrophobic residues L213, C423, M375, Y409, F419 and F424, as well as the backbones of charged residues E215, K216, E420 and K537. The amino group at one side of phenamacril, which is protonated at a neutral or weakly alkaline pH (crystals formed at pH 9), forms charge interaction with the side chain of D540, while the cyanide group at the other side of phenamacril forms a hydrogen bond with the side chain of FgMyoI S217 (Fig 2D).

**Fig 3:**
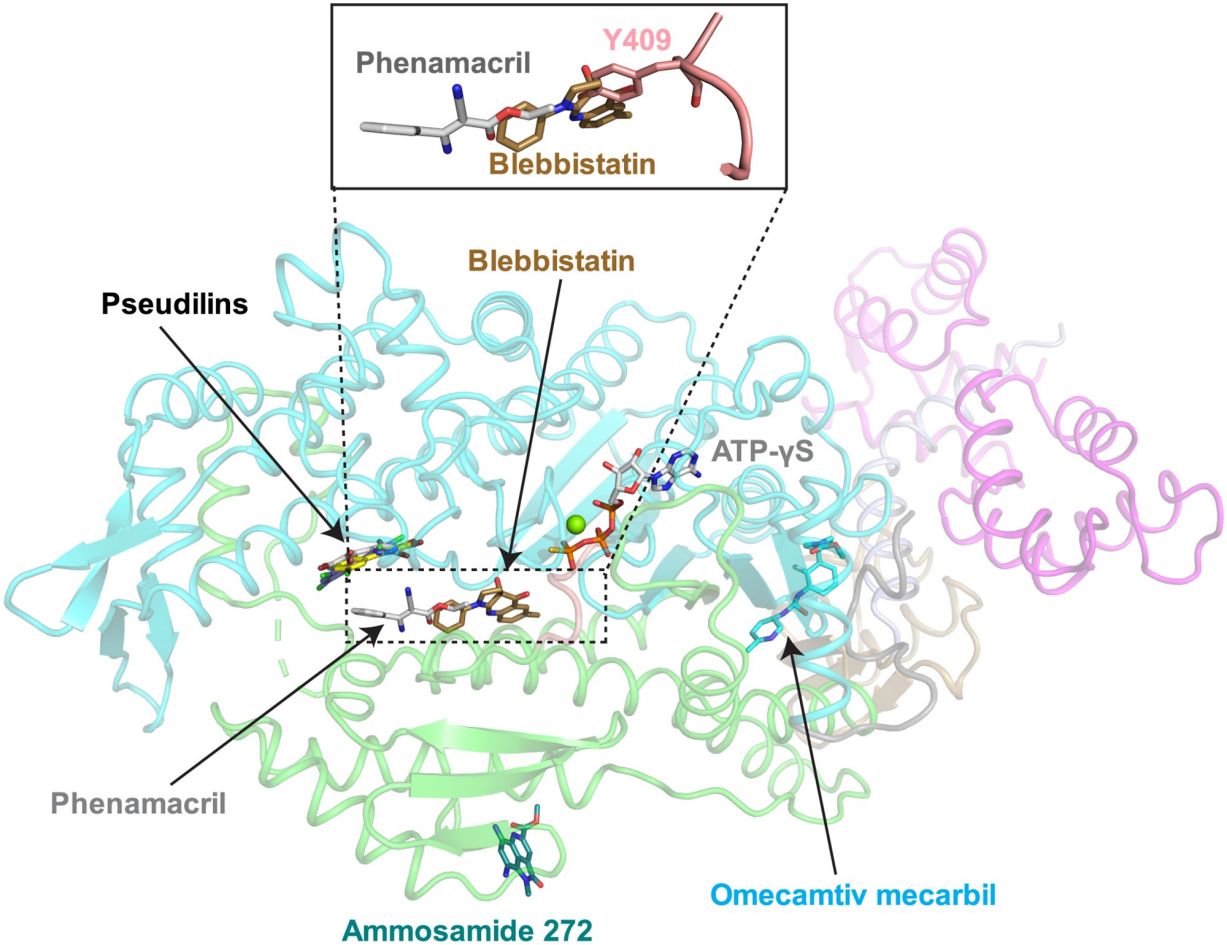
Allosteric myosin motor domain binding sites. Allosteric modulators of myosin motor domains overlaid onto the structure of FgMyoI. Switch 2 is shown in pink. Myosin inhibitors: Pentachloropseudilin (PDB 2XEL [18]), pentabromopseudilin (PDB 2JHR [24]), blebbistatin (PDB 1YV3 [37]), tribromodichlorpseudilin (PDB 2XO8 [38]), ammosamide 272 (PDB 4AE3); positive cardiac inotrope: omecamtiv mecarbil (PDB 4PA0 [39]). The insert illustrates the steric clash between Switch 2 Y409 and blebbistatin.

Allosteric myosin inhibitors and activators are important tools to analyze the complex allosteric communication during the myosin kinetic cycle and the position of four allosteric binding sites have been identified in myosin (Fig 3) [18, 24, 37–39]. Of these, the binding sites for the myosin II inhibitor blebbistatin [37] and the family of pseudilin inhibitors are located within the actin-binding cleft. The pocket occupied by phenamacril has not been previously described and is adjacent to the blebbistatin binding site. Note that the presence of the bulky tyrosine residue (Y409 in FgMyoI) found in the Switch 2 loop of class I myosin (instead of an alanine in Myo2) is not compatible with blebbistatin binding, which could explain why this drug is not efficient against FgMyoI (see Fig 3 insert).

The 17 residues that directly interact with phenamacril include five of the six residues whose mutations have been described to cause high or moderate phenamacril resistance mutations in the myosin I motor domains of *F*. *graminearum* [4] and/or *F*. *asiaticum* [33] (K216E/R, S217P/L, E420K/G/D, I424R, and A577G) (Table 1, Fig 2D and S3 Fig). The sixth resistance-conferring residue, S418, also lines the binding pocket, but does not contact phenamacril directly. Instead, S418 stabilizes the phenamacril-interacting residues F419, K537, and especially E420 (Fig 2D).

**Fig 4:**
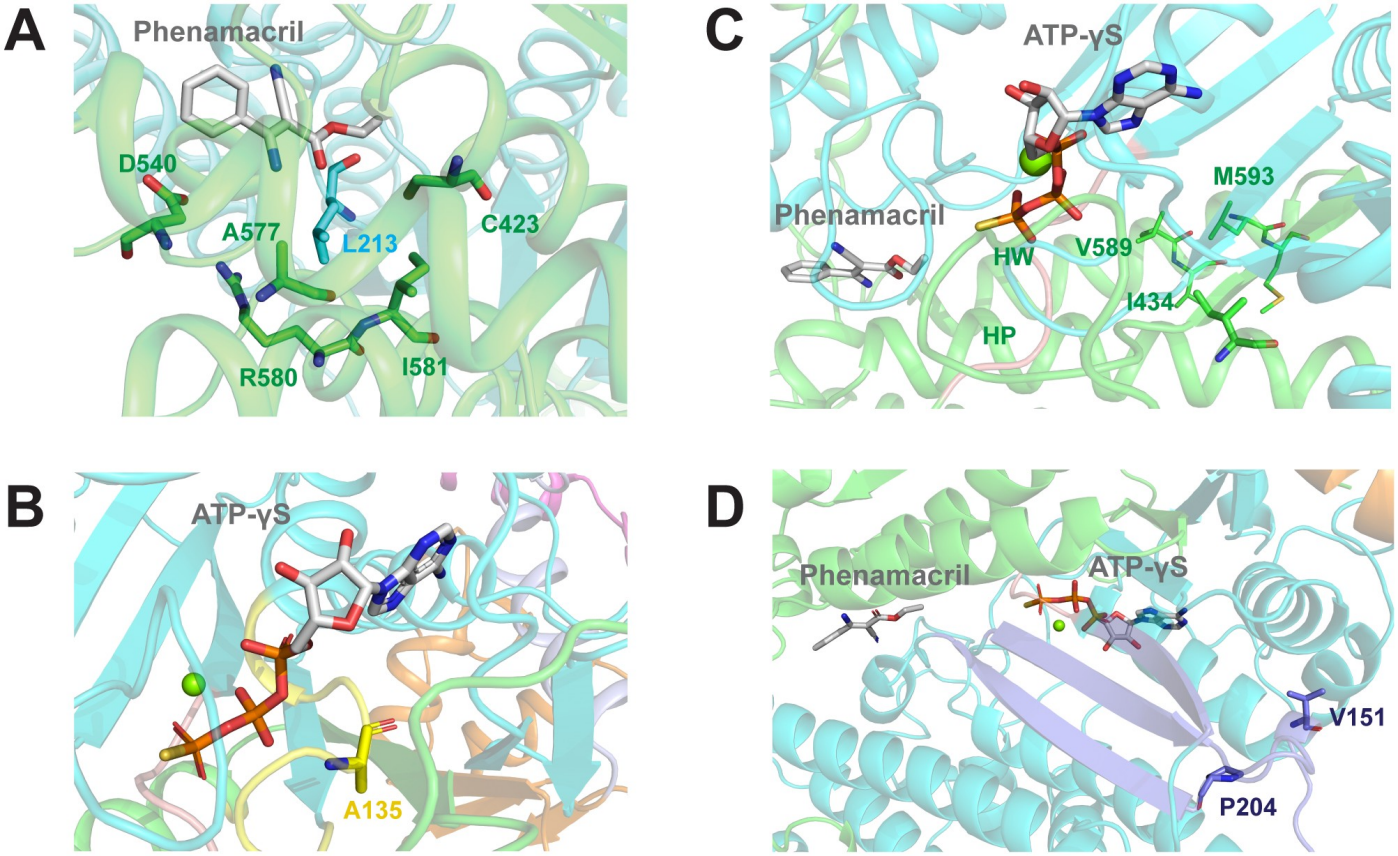
Localization of mutant residues that cause mild phenamacril resistance. (A) A577, R580, and I581 are pocket residues. A577 directly interacts with phenamacril, whereas R580 and I581 interact with pocket residues L213 from U50 (cyan) and C423, D540, and A577 from L50 (green). (B) A135 is a P-loop (yellow) residue of the ATP-pocket that interacts with the α- and β-phosphates of ATP. (C) I434 of helix HP (helix 12) interacts with L588, V589, L592, and M593 of helix HW (helix 19). These interactions likely determine the orientation of the N-terminus of HP, which leads into Switch 2 and directly interacts with phenamacril. (D) V151 and P204 are residues in the Transducer (purple), which communicates conformational changes between motor and converter domains. The Transducer consists of the last three β-strands of the U50 β-sheet and Loop 1 (flanked by V151) and the P204-containing β-bulge.

An additional seven mutations cause mild phenamacril resistance. They include i) an additional mutation in A577 (A577T; Fig 4A and S4 Fig), ii) mutations in two phenamacril pocket-stabilizing residues (R580G/H and I581F; Fig 4A and S4 Fig), iii) one mutation in the ATP-binding pocket (A135T; Fig 4B and S5 Fig), iv) one mutation in the phenamacril- and Switch 2-interacting helix HP (I434M; Fig 4C and S6 Fig), and v) two mutations (V151M, P204S) in the transducer that links the conformational changes between motor and converter domains (Fig 4D and S7 Fig).

### Mutational analysis of the phenamacril-binding pocket

**Fig 5:**
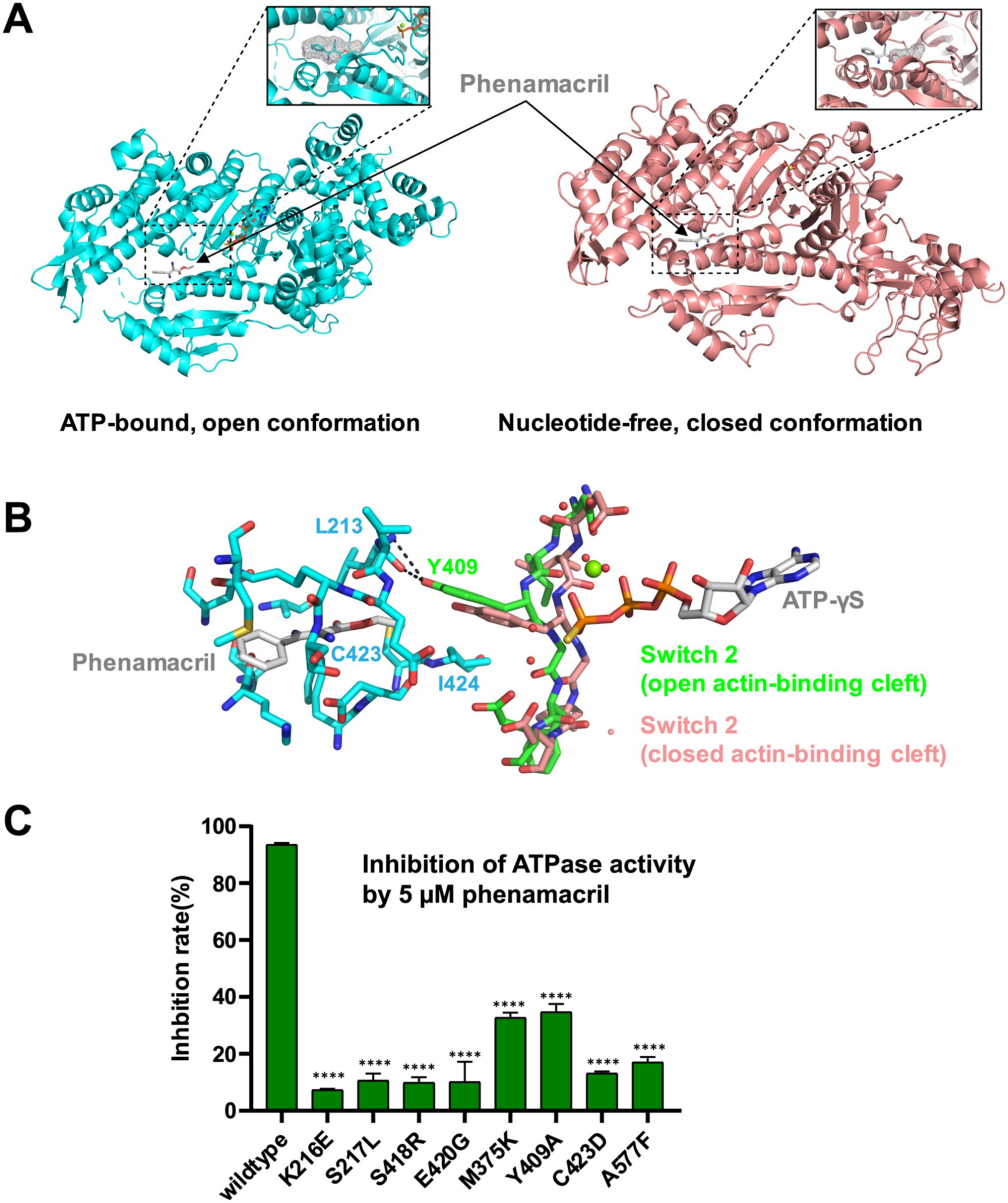
Phenamacril blocks closure of the actin-binding cleft. (A) Side-by-side structural alignment of the myosin motor domain in closed (ATP-γS/phenamacril-bound FgMyoI) and open (nucleotide-free chicken myosin V) conformation overlaid with the position of phenamacril from the FgMyoI structure. The inserts show the phenamacril pocket as mesh in the open conformation and the corresponding pocket in the closed conformation. (B) Switch 2 Y409 binds the phenamacril pocket. Fg Switch 2 in the open (pre-powerstroke) conformation is shown in green, the Switch 2 conformation of chicken myosin V in closed conformation is shown in pink. (C) Phenamacril pocket mutations relieve inhibition of FgMyoI ATPase activity. Reactions contained 0.5 μM myosin and 0.1 μM calmodulin. n = 3, error bars = SD. ****: p-value<0.0001 (One-way ANOVA).

To validate the structure, we generated a series of FgMyoI mutant proteins with replacements of key phenamacril-binding residues including the Switch 2 residue Y409. The F419A mutation destabilized the FgMyoI(1–736) structure as evident by its strongly reduced basal ATPase activity (S2A Fig), reduced purity (S2B Fig), and its poor size exclusion chromatography profile (S2C Fig) and was therefore excluded from further analysis. With the exception of FgMyoI C423D, which was partially destabilized and showed about two-fold reduced basal ATPase activity, all other mutant proteins were stable with ATPase activities similar to or higher than that of the wildtype protein (S2A Fig), yet all exhibited strongly reduced inhibition by phenamacril (Fig 5C).

**Fig 6:**
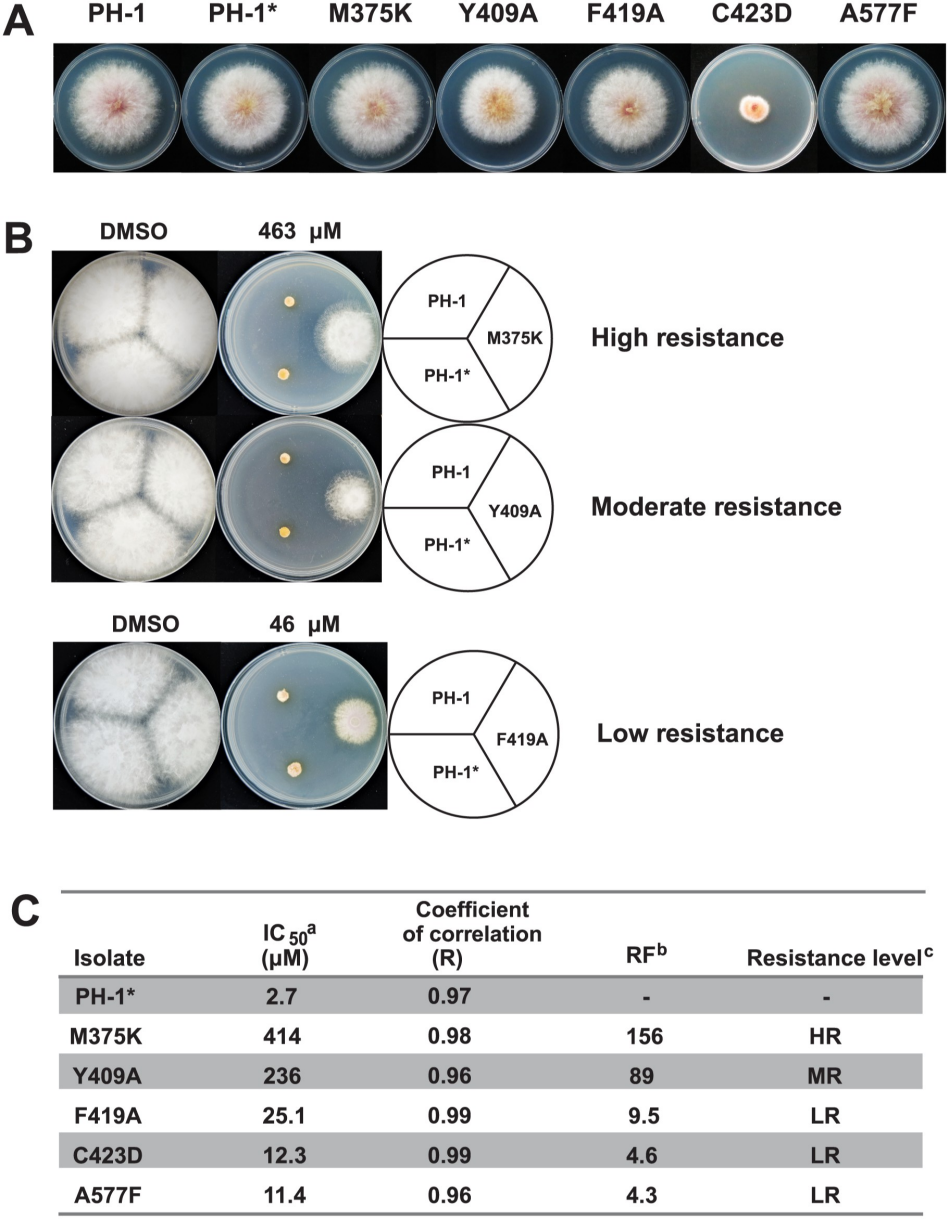
Colony morphology of wild-type (PH-1) and pocket mutant *Fusarium graminearum*. (A) Growth state of *F*. *graminearum* isolates on potato dextrose agarose (PDA) at 25 °C for 3 days. * *F*. *graminearum* PH-1 transformed with hygromycin resistance cassette. (B) Growth state of *F*. *graminearum* isolates on PDA with DMSO and indicated concentration of phenamacril at 25 °C for 3 days. (C) IC_50_ and RF values. ^a^IC_50_ = effective concentration for 50% inhibition. ^b^RF, Resistance factor (ratio of IC_50_ for a resistant isolate relative to IC_50_ for the original isolate. ^c^RF>100 (high resistance, HR);10<RF<100 (intermediate resistance, MR); RF<10 (low resistance, LR).

We further tested whether the mutant proteins would confer phenamacril resistance in *F*. *graminearum*. We used homologous recombination to replace the wildtype myosin-5 gene with the M375K, Y409A, F419A, C423D and A577F variants at their endogenous locus in the wildtype *F*. *graminearum* strain PH-1 (see Methods). All knock-in mutants grew normal-sized mycelia in the absence of phenamacril except the partially unstable C423D (S2C Fig), whose growth was severely defective (Fig 6A). While the rank order differs from that for the inhibition of the ATPase activity of recombinant FgMyoI, all endogenously expressed mutant genes showed phenamacril resistance to various degrees, with the M375K and Y409A mutants exhibiting the highest resistance levels (Fig 6B and 6C).

### Phenamacril likely blocks closure of the actin-binding cleft and may stabilize Switch 2 in the pre-powerstroke conformation

**Fig 7:**
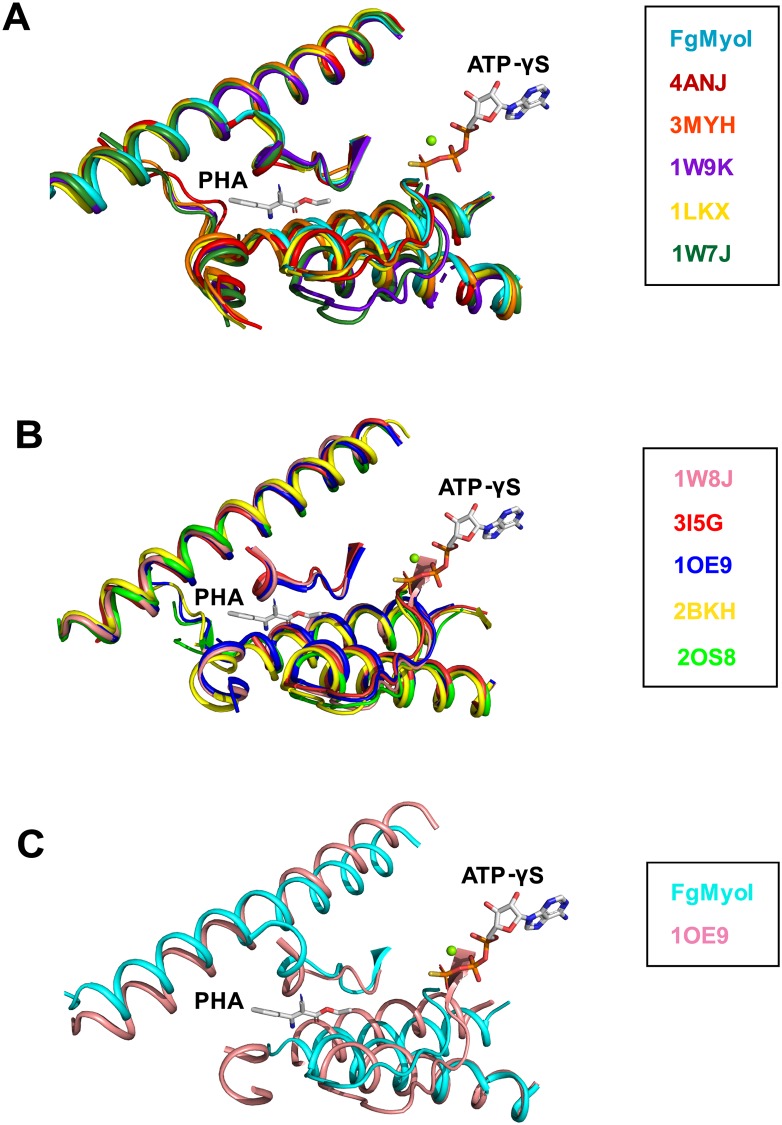
Alignment of myosin domains in nucleotide-bound and nucleotide-free states. (A) Alignment of FgMyoI with five structures of myosin motor domains in nucleotide-bound state with relatively more open actin-binding clefts. (B) Alignment of five structures of myosin motor domains in nucleotide-free state with closed actin-binding clefts. The positions of phenamacril (PHA) and ATP-γS from FgMyoI are shown for orientation. Parts of the motor domains that are not close to the phenamacril-binding pocket have been removed for clarity. (C) Close-up of structure overlay of FgMyoI with that of chicken myosin in nucleotide-free, closed cleft conformation. (4ANJ [44]/2BKH [45]: *Sus scrofa* class 6 myosin; 3MYH [46]/1W9K: *Dictyostelium discoideum* class 2 myosin; 1LKX [36]: *Dictyostelium discoideum* class 1 myosin; 1W7J [40]/1W8J [40]/1OE9 [41]: *Gallus gallus* class 5 myosin; 3I5G [42]: *Doryteuthis pealeii* class 2 myosin; 2OS8 [42]: *Placopecten magellanicus* class 2 myosin).

How does phenamacril inhibit the FgMyoI ATPase activity? Phenamacril binds outside of the catalytic center and clearly does not block ATP binding. Also, ATP, Mg^2+^, and water molecules are correctly positioned for ATP hydrolysis (Fig 2D). Consistently, recent detailed biochemical analysis has demonstrated that phenamacril does not change the affinity of FgMyoI for ATP, yet reduces its affinity for actin at least five-fold [27]. ATP is rapidly hydrolyzed in the catalytic site in the absence of actin, but the release of the hydrolyzed phosphate is slow as it requires rearrangement of the phosphate-binding Switch1 and Switch 2 loops and requires actin binding for acceleration [10]. Weak electrostatic actin binding partially closes the actin-binding cleft, which is thought to stabilize a switch loop movement that creates a tunnel for phosphate release. Phosphate release in turn is linked to lever arm movement (power stroke) and full cleft closure and actin binding, although the exact order of these events remains controversial [9, 10] (Fig 1A). Structural alignment of phenamacril-bound FgMyoI with the motor domain of chicken myosin V in nucleotide-free, actin binding cleft-closed state [40] illustrates that in the absence of further substantial rearrangements phenamacril binding sterically interferes with cleft closure (Fig 5A). Phenamacril occupies a pocket of 238 Å^3^ in the FgMyoI open cleft structure, whereas the pocket in the myosin V closed cleft structure [41] is much smaller with a volume of only 61 Å^3^, which is too small to accommodate phenamacril. While there is some variability in the degree of actin-binding cleft opening and closing in nucleotide- or nucleotide analog-bound structures and nucleotide-free structures [40, 42], respectively, structural alignment of multiple myosins in both states show that cleft closure sterically interferes with phenamacril binding independent of myosin subtype (Fig 7). While phenamacril could affect more than one step in the myosin/actin cycle, its binding therefore likely arrests myosin in the “open”, pre-powerstroke state, similar to the proposed mechanism of myosin II inhibition by blebbistatin [37, 43]. Moreover, Switch 2 Y409 in the pre-powerstroke conformation directly binds phenamacril as well as the phenamacril-pocket residues L213, C423, and I424, but cannot do so in the superimposed myosin V closed actin-binding cleft conformation (Fig 5B). Phenamacril binding of Y409 may thereby further stabilize the Switch 2 pre-powerstroke conformation, providing a conformational link between pocket occupancy, Switch 2 conformation, and lever arm movement.

### M375 is a key phenamacril sensitivity determinant

**Fig 8:**
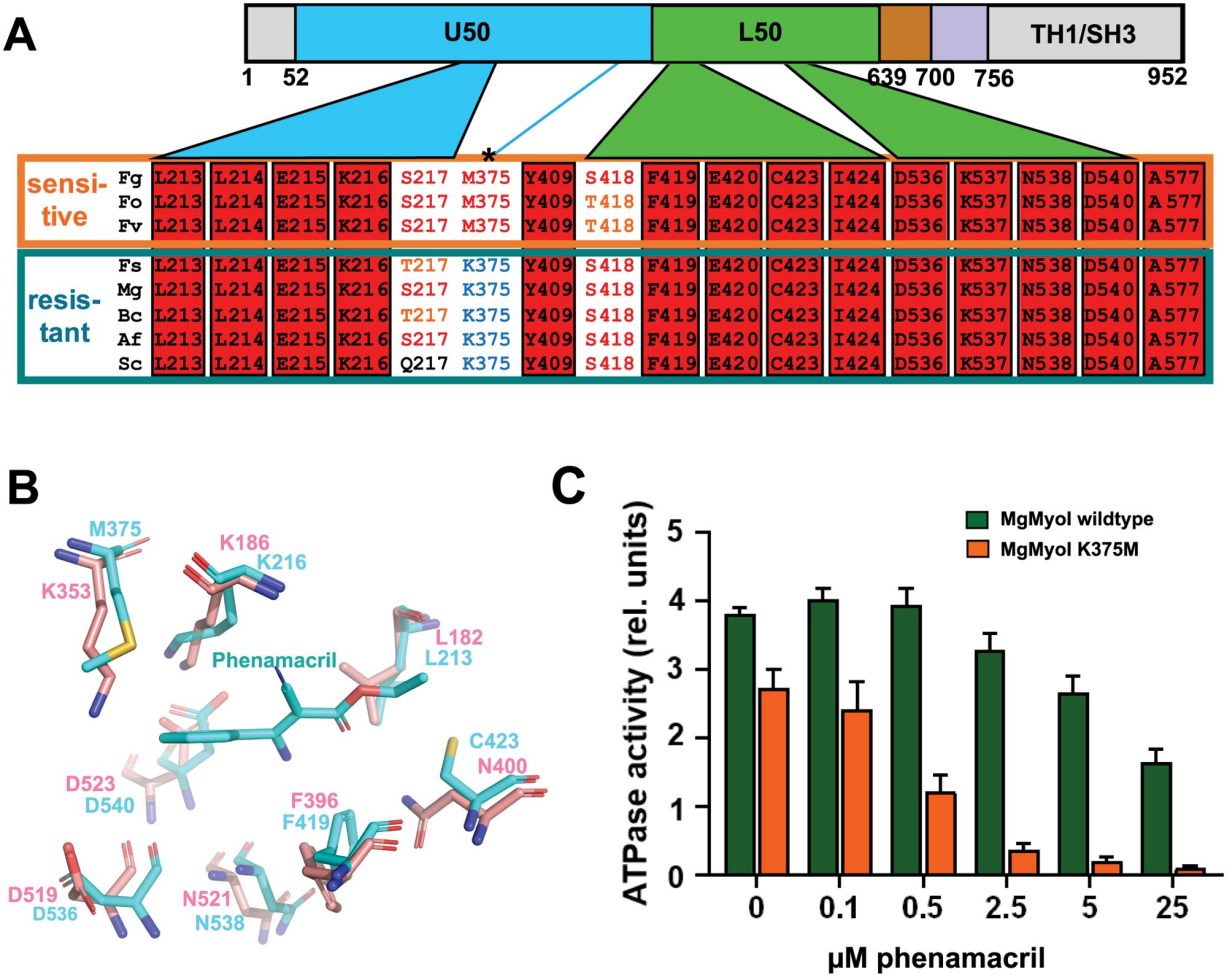
M375 is a major phenamacril specificity determinant. (A) Conservation among the phenamacril pocket residues in myosin I motor domains from phenamacril-sensitive strains (Fg, Fo, Fv) and phenamacril-insensitive strains (Fs, Mg, Bc, Af, Sc). Fg: *Fusarium graminearum*, Fo: *F*. *oxysporin*, Fv: *F*. *verticillioides*, Fs: *F*. *solani*, Mg: *Magnaporthe grisea*, Bc: *Botrytis cinerea*, Af: *Aspergillus flavus*, Sc: *Saccharomyces cerevisiae*. (B) Overlay of FgMyoI phenamacril pocket residues (cyan) and their corresponding residues from *Dictyostelium discoideum* myosin I (pink, PDB 1LKX [36]). For clarity, only those residues are shown that have different side chain positions among the two myosins. (C) Mutation of MgMyoI K375 to M confers phenamacril sensitivity. ATPase activity (relative luminescence units) of purified wildtype and K375M MgMyoI/CaM in the presence of the indicated concentrations of phenamacril; n = 3, error bars = SD.

Identification of the 18 phenamacril pocket residues (17 phenamacril-binding residues plus the pocket-stabilizing S418) allowed us to compare their conservation across phenamacril-sensitive and -resistant *Fusarium* species as well as species from other fungal genera. As shown in Fig 8A, S1 and S2 Tables, pocket residues are highly conserved with only three residues showing sequence variations across several species: S217, M375, and S418. Of these, only M375 shows replacements against a dissimilar amino acid, lysine, and the M375K mutation reduced inhibition of FgMyoI ATPase activity (Fig 5C) and conferred phenamacril resistance in *Fusarium* (Fig 6C). Moreover, the motor domains of all analyzed phenamacril-resistant species have a lysine at this position whereas it is a methionine in all analyzed sensitive species (Fig 8A, S2 Table). We structurally aligned the pockets of FgMyoI and myosin 1E from *Dictyostelium discoideum* (DdMyoI) [36]. The DdMyoI and FgMyoI pockets differ only in two residues: M375 is replaced by K353 and C423 by N400 (Fig 8B), respectively. While side chains of four of the identical residues have rearranged in phenamacril-bound FgMyoI to more favorably interact with the ligand (L213, K216, F419, and D540), the main difference is M375/K353 (Fig 8B). The exchange against K353 both eliminates extensive Van-der-Waals interactions with the phenyl group of phenamacril and adds an unfavorable charge (ε-amino group in 2.9 Å distance from the phenyl group), providing a structural rationale for the effect of M375K on phenamacril specificity. The C423/N400 exchange further reduces Van-der-Waals interactions, but the N400 carboxamide can form a compensating hydrogen bond with phenamacril’s carbonyl group. Together, this suggests that M375 is the key determinant for phenamacril specificity within the binding pocket. To provide further experimental evidence for the importance of M375 for phenamacril sensitivity, we tested whether we could also mutationally convert a phenamacril resistant myosin to a phenamacril sensitive one. We generated recombinant wildtype (K375) and K375M myosin I protein from *Magnaporte grisea* (MgMyoI, a fungus with a very weak phenamacril response). As shown in Fig 8C, the MgMyoI K375M mutation conferred strongly increased phenamacril sensitivity. Together, this provides comprehensive support for the important role of M375 in phenamacril selectivity.

## Discussion

**Fig 9:**
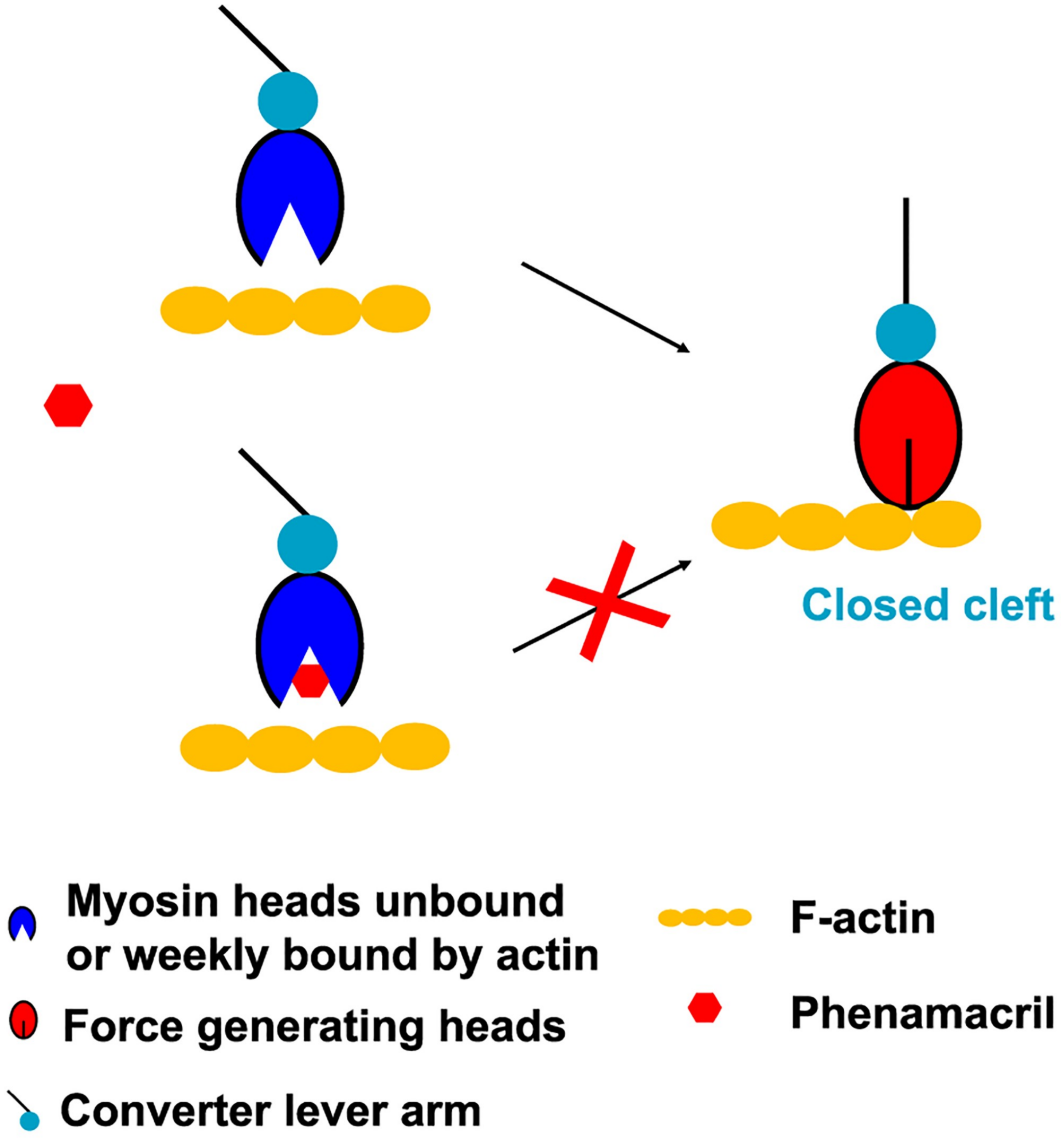
Simplified model of the proposed phenamacril resistance mechanism. Binding of phenamacril in the actin-binding cleft blocks cleft closure. The open cleft myosin motor domain is in equilibrium between free and phenamacril-bound states.

Phenamacril is a highly specific myosin inhibitor. This high specificity is exploited for its use as selective fungicide in crop fields, but the molecular basis for its high specificity has been unknown. Our structure of phenamacril-bound FgMyoI(1–736) has illustrated phenamacril’s likely primary mechanism of action, arrest of the motor domain in the pre-power stroke conformation by blocking closure of the actin-binding cleft (Fig 9). The structure has identified the detailed interaction of phenamacril with 18 amino acids that line the novel allosteric pocket in the actin-binding cleft, and all known high and moderate phenamacril resistance mutations affect six of these pocket residues. Based on the structure, we have generated additional pocket residue mutations that confer partial or full resistance to inhibition of the ATPase activity of FgMyoI.

We have identified Y409 and M375 as two key residues. Y409, the central amino acid in myosin I Switch 2, binds phenamacril and the phenamacril-binding pocket only in the open conformation, thereby conformationally linking pocket occupancy to the Switch 2 pre-power stroke conformation. Y409 occupies the variable (S, A, Y, or F) residue of the Switch 2 consensus sequence (D-I-S/A/Y/F-G-F-E). This residue is a tyrosine in all class I myosins that we have analyzed, and thus contributes to myosin subtype selectivity. While most pocket residues are highly conserved, M375 is an atypical residue. In an alignment of 267 myosins from diverse species less than 3% have a methionine in the corresponding position and none of them includes a myosin-1 from a non-*Fusarium* species [3]. However, M375 is found in all analyzed myosin I motor domains from phenamacril-sensitive *Fusarium* species. We have directly confirmed the key role of M375 in phenamacril selectivity by demonstrating that its mutation to K confers phenamacril resistance in *F*. *graminearum* and mutation of K375 to M confers sensitivity in *M*. *grisea*.

To our knowledge, the structure of FgMyoI(1–736) is the first solved myosin structure of a plant pathogen. In addition to plant pathogenic species, a number of *Fusarium* species are also causal agents of fungal keratitis in humans [47–50]. A recent analysis has shown that phenamacril is not only effective against *Fusarium* species in crop fields, but also against *F*. *oxysporum* 32931, a human-pathogenic *Fusarium* strain that is a cause of fungal keratitis (IC_50_ of 0.3 μg/mL, compared to 0.42 μg/mL and 0.44 μg/mL for *F*. *graminearum* and *F*. *asiaticum*, respectively [30]). Fungal keratitis is a leading cause of ocular morbidity [47], and more than 50% of fungal keratitis are caused by *Fusarium* species [51]. Of these, only *F*. *oxysporum* has been tested for phenamacril susceptibility to date. Since more species show resistance to antifungals, including natamycin, the frontline drug for the treatment of infections by filamentous fungi, it is also urgent to develop new drugs against human-pathogenic *Fusarium* species [47].

In conclusion, the structure has elucidated the structural basis of phenamacril action, resistance and selectivity. It further provides a framework to guide the development of novel fungicides that bind the pocket with higher affinity to overcome resistance to the single pocket residue mutations that compromise phenamacril’s long-term use.

## Materials and methods

### Protein preparation

The coding sequence of FgMyoI(1–736) with C-terminal His8-tag was amplified from cDNA of *F*. *graminearum* strain PH-1. The FgCaM coding sequence was synthesized by Eurofins. The FgMyoI(1–736)-His8 coding region was cloned into pFastBac^™^. Recombinant baculovirus was prepared using the Baculovirus/Sf9 Expression System (Life Technology). FgMyoI(1–736) and FgCaM were co-expressed by co-transfecting 1 L Sf9 insect cells at a density of 280×10^4^ cells with 15 mL FgMyoI(1–736) P1 virus and 5 mL FgCaM P1 virus. Transfected Sf9 cells were incubated at 28°C and 110 rpm for 48 hours. Cell pellets were harvested at 1,328 × g in a H12000 rotor for 20 minutes and resuspended in TBS buffer with 0.4 mM EGTA. Then cell pellets were lysed by sonication in 100 mL lysis buffer [50 mM Tris-HCl pH 8.4, 300 mM NaCl, 10% glycerol, 1 mM EGTA, 0.02% NaN_3_, 2.5 mM ATP, 5 mM of 2-mercaptoethanol, 0.2 mg/mL trypsin inhibitor (Sigma), 0.5% Triton X-100, 1 tablet EDTA-free protease inhibitor cocktail (Roche)]. After centrifugation at 186,000 × g in a Type 45 Ti rotor for 45 minutes, the supernatant was incubated with 5 mL nickel sepharose beads (GE Healthcare) on a rotating wheel at 4°C for 2 h. Then nickel beads were transferred to a gravity flow column and washed with 2 column volumes of lysis buffer, followed by 5 column volumes of buffer A (50 mM Tris-HCl pH 8.4, 200 mM NaCl, 25 mM imidazole, 0.1 mM dithiothreitol, 10% glycerol). The FgMyoI(1–736)/FgCaM complex was eluted with 5 column volumes of buffer B (50 mM Tris-HCl pH 8.4, 200 mM NaCl, 500 mM imidazole, 0.1mM EGTA, 0.1 mM dithiothreitol, 10% glycerol). The complex was further purified by chromatography through a Superdex 200 Increase 10/300 GL gel filtration column (GE Healthcare) in 20 mM Tris-HCl pH 8.4, 200 mM NaCl, 10% glycerol and concentrated to 9 mg/mL.

To generate FgCaM separately to titrate into FgMyoI preparations, FgCaM was expressed in and purified from *Escherichia coli* BL21 (DE3). FgCaM was expressed as fusion protein with a cleavable N-terminal His6Sumo tag from a modified pETDuet1-SUMO (LifeSensors) expression vector. BL21(DE3) cells transformed with the expression plasmid were grown in 2 L LB broth at 16°C to an OD_600_ of ∼1.0 and were induced with 0.1mM isopropyl β-D-1-thiogalactopyranoside (IPTG) for 16 h. Cells were harvested, resuspended in 150 mL extract buffer [25 mM Tris (pH 8.4), 150 mM NaCl, 1mM dithiothreitol and 10% (vol/vol) glycerol] and were passed two times through a French press with pressure set at 1,000 Pa. The lysate was centrifuged at 34,572 × g in a F21-8×50y rotor for 1 h, and the supernatant was loaded on a 5-mL HisTrap HP column (GE Healthcare). The column was washed with 100 mL of buffer A [25 mM Tris (pH8.4), 150 mM NaCl, 25 mM imidazole, 1 mM dithiothreitol and 10% (vol/vol) glycerol] and was eluted with 100 mL of 100% buffer B [25 mM Tris (pH8.4), 150 mM NaCl, 500 mM imidazole, 1 mM dithiothreitol and 10% (vol/vol) glycerol]. The eluted His6Sumo-FgCaM was dialyzed against extract buffer and cleaved overnight with SUMO protease (ULP1) at a protease/ protein ratio of 1:500 at 4 °C. The cleaved His6Sumo tag was removed by passing through a 5-mL HisTrap HP column, and the protein was further purified by chromatography through a HiLoad Superdex 16/600 Superdex 200 gel filtration column (GE) in 20 mM Tris (pH 8.4), 200 mM NaCl, 1 mM dithiothreitol and 10% (vol/vol) glycerol.

MgMyoI(1–739) with C-terminal His8-tag was amplified from cDNA of *M*. *grisea* strain Guy11. The MgMyoI(1–739)-His8 coding region was cloned into pFastBac. Recombinant baculovirus was prepared using the same Baculovirus/Sf9 Expression System. MgMyoI(1–736) and FgCaM were co-expressed and purified following the same method as for the FgMyoI(1–736) wildtype protein above.

### ATPase assays

ATPase assays were performed using the ADP-Glo Kinase Assay (Promega) in 1× Kinase Reaction Buffer A (40 mM Tris pH 7.5, 20 mM MgCl_2_ and 0.1 mg/mL BSA) containing 0.5 mM ATP. For ATPase activity test of FgMyoI(1–736), reactions were started by the addition of 0.5 μM FgMyoI(1–736) protein with or without 0.1 μM FgCaM. For inhibition of FgMyoI(1–736) by phenamacril, reactions containing 0.5 μM FgMyoI(1–736) and 0.1 μM FgCaM were started by the addition of 0.5 mM ATP in the presence of indicated concentrations of phenamacril diluted in 1× Kinase Reaction Buffer A. After incubation at 25°C for 30 minutes, 5 μL of ADP-Glo Reagent was added to stop the ATPase reaction and deplete unconsumed ATP, followed by a 40-minute incubation at 25 °C. Then 10 μL of Kinase Detection Reagent was added to convert ADP to ATP, followed by incubation with luciferase and luciferin for 40 minutes at room temperature to detect ATP by measuring the luminescence with a plate-reading luminometer. For ATPase activity test and inhibition of FgMyoI(1–736) mutant proteins, MgMyoI(1–739) wildtype and mutant K375M proteins, the same methods were followed as described above. All the experiments were conducted using three biological replicates.

### Crystallization

The FgMyoI(1–736)-FgCaM complex was incubated with phenamacril (kindly provided by the Institute for the Control of Agrochemicals, Ministry of Agriculture (ICAMA), Hangzhou, China) at a 1:5 molar ratio in the presence of 2 mM MgCl_2_ and 2 mM adenosine 5'-(γ-thio)-triphosphate (ATP-γS; Cayman Chemical) (final concentrations) for 1 h on ice. After centrifugation at 16,000 g in a F45-24-11 rotor for 10 min, FgMyo-FgCaM-phenamacril crystals were grown in sitting drop format containing 0.2 μL of 9 mg/mL purified FgMyo-FgCaM protein, 2 mM ATP-γS, 2 mM MgCl_2_ and 0.2 μL of well solution containing 0.1 M BIS-TRIS propanol, pH 9, and 30% (w/v) polyethylene glycol 6,000 for 2 days at 20 °C. All crystals were serially transferred to the well solution with 20% (v/v) ethylene glycol before flash freezing in liquid nitrogen.

### Data collection, structure determination and analysis

The crystals of FgMyoI(1–736)/FgCaM/phenamacril formed in the P212121 space group. The datasets were collected with an EIGER 9M pixel array detector at the ID-D line of sector 21 of the Advanced Photon Source at Argonne National Laboratory (Argonne, IL). The data was indexed to 2.65 Å with XDS [52] and scaled with AIMLESS in ccp4 package (http://www.ccp4.ac.uk). The CCP4 program PHASER was used for molecular replacement, with the crystal structure of human Myosin 1c (PBD code: 4BYF) [53] as a search model. The calmodulin model was built based on election density with a homology model from a rat calmodulin (PDB code: 5WBX) [54]. The initial model was manually built in COOT [55] and refined with the PHENIX program phenix.refine [56]. All figures were prepared using PyMOL (DeLano Scientific, San Carlos, CA, http://www.pymol.org) (Table 2). The volume of phenamacril binding pocket of FgMyoI was calculated with VOIDOO [57].

### Mutagenesis of FgMyoI and MgMyoI

FgMyoI(1–736) mutant constructs and MgMyoI(1–739) mutant K375M construct were made using the QuickChange Method (Agilent) with Pfast-FgMyoI(1–736)-H8 and pFast-MgMyoI(1–739)-H8 as template. Mutations for all plasmid constructs were confirmed by sequencing. The expression and purification of FgMyoI(1–736) mutant proteins and MgMyoI(1–739) mutant protein followed the same method as for the wildtype protein above.

### FgMyoI knock-in mutagenesis and fungicide susceptibility testing

FgMyoI mutant replacement cassettes for homologous recombination were generated by double-joint PCR [58] as described previously [4]. Briefly, the myosin-5 gene fragment of *F*. *graminearum*, a hygromycin resistance gene hygromycin B phosphotransferase (HPH) under control of the *Aspergillus nidulans trpC* promoter, and the upstream flanking sequence of myosin-5 were mixed and incubated with polymerase to generate a fusion product by primer extension in the absence of external primers. The product was used as DNA template to amplify a 6 kb DNA fragment that was transformed into protoplasts of the wild-type strain PH-1. The protoplast preparation and transformation of *F*. *graminearum* were performed as previously described [59]. Transformants were selected on medium containing 100 mg/mL hygromycin B and were verified by PCR and DNA sequencing of the entire myosin coding region. Single-copy insertion of the cassette was confirmed by Southern blotting of genomic DNA digested with *SacI* [4]. Susceptibility to phenamacril was assessed for all transformants as described previously [4] and three biological replicates were used for each strain and each experiment was repeated three times independently.

## Supporting information

S1 Fig Related to Fig 2. FgMyoI/FgCaM structure. (A) FgMyoI-phenamacril-FgCaM complex structure with 2Fo-Fc map contoured at 1 σ for the myosin domains, and contoured at 0.7 σ for FgCaM. (B) Cartoon presentation of the FgMyoI-phenamacril-FgCaM complex structure with B factors of the structure depicted in a rainbow color code from blue for B factors around 40 Å^2^, to red for B factors about 250 Å^2^. (C) Structural comparison of FgMyoI-bound FgCaM with calmodulins that bind myosin Ic (left panel, PDB: 4R8G) and myosin 5a (right panel, PDB: 4ZLK), respectively. The RMSD between FgMyoI-bound FgCaM and myosin Ic-bound calmodulin, and myosin 5a-bound calmodulin are 6.1, and 6.9 Å, respectively. FgCaM and FgMyoI-bound FgCaM is colored in magenta; myosin Ic-bound calmodulin, in gray; and myosin 5a-bound CaM in yellow. Both lever arms of myosins Ic and 5a are omitted for clarity, except the IQ1 helix of FgMyoI, which is colored in blue.
(TIF)

S2 Fig Related to Fig 5C. Activity and SEC profiles of MyoI mutant proteins in the absence of phenamacril. (A) ATPase activity (500 nM FgMyoI+/-100 nM CaM); n = 3, error bars = SD. (B) Protein purity (SDS PAGE). (C) Size exclusion chromatograms of wildtype and mutant FgMyoI. (D) Size exclusion chromatograms of wildtype and mutant MgMyoI. SDS PAGE inserts in the SEC panels show the proteins from the two top elution fractions. Note that FgMyoI F419A, E420G, and C423D are largely aggregated and co-purified calmodulin is not or only poorly visible. AP: Aggregation peak.
(TIF)

S3 Fig Related to Fig 2D and Table 1. Pockets of phenamacril-resistance mutants. (A) K216 mutations. (B) S217 mutations. (C) E420 mutations. (D) I424 mutation. (E) A577 mutation. Resistance mutations were modeled in the phenamacril binding site with wildtype and mutant pockets shown in the same orientation next to each other.
(TIF)

S4 Fig Related to Fig 4A. Stereo image of Fig 4A. (TIF)

S5 Fig Related to Fig 4B. Stereo image of Fig 4B. (TIF)

S6 Fig Related to Fig 4C. Stereo image of Fig 4C. (TIF)

S7 Fig Related to Fig 4D. Stereo image of Fig 4D. (TIF)

S1 Table Alignment of the FgMyoI (1–736) sequence with sequences of myosins with solved motor domain structures. *Dictyostelium discoideum* class 1 myosin: PDB 1LKX; *Rattus norvegicus* class 1 myosin: PDB 5V7X; *Argopecten irradians* class 2 myosin: PDB 1DFL; *Placopecten magellanicus* class 2 myosin: PDB 2EC6; *Doryteuthis pealeii* class 2 myosin: PDB 3I5G; *Bos Taurus* class 2 myosin: PDB 5N6A; *Dictyostelium discoideum* class 2 myosin: PDB 2Y8I; *Gallus gallus* class 5 myosin: PDB 1OE9; *Mus musculus* class 5 myosin: PDB 4ZLK; *Homo sapiens* class 10 myosin: PDB 5KG8; *Sus scrofa* class 6 myosin: PDB 4ANJ.
(PDF)

S2 Table Sequence alignment of the myosin I motor domains of phenamacril-sensitive (red box) and phenamacril-resistant species. Pocket residues are labeled with variable pocket residues highlighted in green. F.: *Fusarium*; M.: *Magnaporthe*; B.: *Botrytis*; A.: *Aspergillus*; S.: *Saccharomyces*; H.: *Homo*; D.: *Dictyostelium*; *: Amino acid whose mutation cause high or medium resistance to phenamacril.
(PDF)

## References

[1] Goswami RS, Kistler HC. Heading for disaster: *Fusarium graminearum* on cereal crops. Mol Plant Pathol. 2004;5(6):515–25. Epub 2004/11/01. 10.1111/j.1364-3703.2004.00252.x . 20565626

[2] Xu X, Nicholson P. Community ecology of fungal pathogens causing wheat head blight. Annu Rev Phytopathol. 2009;47:83–103. Epub 2009/04/24. 10.1146/annurev-phyto-080508-081737 . 19385728

[3] Foth BJ, Goedecke MC, Soldati D. New insights into myosin evolution and classification. Proc Natl Acad Sci USA. 2006;103(10):3681–6. Epub 2006/03/01. 10.1073/pnas.0506307103 . 16505385 PMC1533776

[4] Zheng Z, Hou Y, Cai Y, Zhang Y, Li Y, Zhou M. Whole-genome sequencing reveals that mutations in myosin-5 confer resistance to the fungicide phenamacril in *Fusarium graminearum*. Sci Rep. 2015;5:8248 10.1038/srep08248 https://www.nature.com/articles/srep08248#supplementary-information. 25648042 PMC5389027

[5] Ross JL, Ali MY, Warshaw DM. Cargo transport: molecular motors navigate a complex cytoskeleton. Curr Opin Cell Biol. 2008;20(1):41–7. Epub 2008/01/30. 10.1016/j.ceb.2007.11.006 . 18226515 PMC2688467

[6] Hartman MA, Spudich JA. The myosin superfamily at a glance. J Cell Sci. 2012;125(Pt 7):1627–32. Epub 2012/05/09. 10.1242/jcs.094300 . 22566666 PMC3346823

[7] Schröder RR, Manstein DJ, Jahn W, Holden H, Rayment I, Holmes KC, et al Three-dimensional atomic model of F-actin decorated with Dictyostelium myosin S1. Nature. 1993;364(6433):171–4. 10.1038/364171a0 8321290

[8] Uyeda TQ, Abramson PD, Spudich JA. The neck region of the myosin motor domain acts as a lever arm to generate movement. Proc Natl Acad Sci U S A. 1996;93(9):4459–64. 10.1073/pnas.93.9.4459 . 8633089 PMC39560

[9] Llinas P, Isabet T, Song L, Ropars V, Zong B, Benisty H, et al How actin initiates the motor activity of myosin. Dev Cell. 2015;33(4):401–12. 10.1016/j.devcel.2015.03.025 25936506 PMC4476657

[10] Houdusse A, Sweeney HL. How myosin generates force on actin filaments. Trends Biochem Sci. 2016;41(12):989–97. 10.1016/j.tibs.2016.09.006 27717739 PMC5123969

[11] Woody MS, Winkelmann DA, Capitanio M, Ostap EM, Goldman YE. Single molecule mechanics resolves the earliest events in force generation by cardiac myosin. Elife. 2019;8 Epub 2019/09/19. 10.7554/eLife.49266 . 31526481 PMC6748826

[12] Sweeney HL, Houdusse A. Myosin VI rewrites the rules for myosin motors. Cell. 2010;141(4):573–82. Epub 2010/05/19. 10.1016/j.cell.2010.04.028 . 20478251

[13] Sweeney HL, Houdusse A. Structural and functional insights into the myosin motor mechanism. Annual Review of Biophysics. 2010;39(1):539–57. 10.1146/annurev.biophys.050708.133751 20192767

[14] Kronert WA, Bell KM, Viswanathan MC, Melkani GC, Trujillo AS, Huang A, et al Prolonged cross-bridge binding triggers muscle dysfunction in a *Drosophila* model of myosin-based hypertrophic cardiomyopathy. ELife. 2018;7:e38064 10.7554/eLife.38064 30102150 PMC6141233

[15] Arjonen A, Kaukonen R, Mattila E, Rouhi P, Högnäs G, Sihto H, et al Mutant p53-associated myosin-X upregulation promotes breast cancer invasion and metastasis. J Clin Invest. 2014;124(3):1069–82. Epub 02/03. 10.1172/JCI67280 . 24487586 PMC3934176

[16] REDowicz MJ. Myosins and deafness. J Muscle Res Cell M. 1999;20(3):241–8. 10.1023/A:1005403725521 10471988

[17] Miyata M, Kishimoto Y, Tanaka M, Hashimoto K, Hirashima N, Murata Y, et al A role for myosin Va in cerebellar plasticity and motor learning: a possible mechanism underlying neurological disorder in myosin Va disease. J Neurosci. 2011;31(16):6067 10.1523/JNEUROSCI.5651-10.2011 21508232 PMC6632970

[18] Chinthalapudi K, Taft MH, Martin R, Heissler SM, Preller M, Hartmann FK, et al Mechanism and specificity of pentachloropseudilin-mediated inhibition of myosin motor activity. J Biol Chem. 2011;286(34):29700–8. 10.1074/jbc.M111.239210 21680745 PMC3191011

[19] Straight AF, Cheung A, Limouze J, Chen I, Westwood NJ, Sellers JR, et al Dissecting temporal and spatial control of cytokinesis with a myosin II inhibitor. Science. 2003;299(5613):1743 10.1126/science.1081412 12637748

[20] Cheung A, Dantzig JA, Hollingworth S, Baylor SM, Goldman YE, Mitchison TJ, et al A small-molecule inhibitor of skeletal muscle myosin II. Nat Cell Biol 2002;4(1):83–8. 10.1038/ncb734 11744924

[21] Higuchi H, Takemori S. Butanedione monoxime suppresses contraction and ATPase activity of rabbit skeletal muscle. J Biol Chem. 1989;105(4):638–43. 10.1093/oxfordjournals.jbchem.a122717 2527229

[22] Green EM, Wakimoto H, Anderson RL, Evanchik MJ, Gorham JM, Harrison BC, et al A small-molecule inhibitor of sarcomere contractility suppresses hypertrophic cardiomyopathy in mice. Science. 2016;351(6273):617–21. Epub 2016/02/26. 10.1126/science.aad3456 . 26912705 PMC4784435

[23] Kawas RF, Anderson RL, Ingle SRB, Song Y, Sran AS, Rodriguez HM. A small-molecule modulator of cardiac myosin acts on multiple stages of the myosin chemomechanical cycle. J Biol Chem. 2017;292(40):16571–7. Epub 2017/08/16. 10.1074/jbc.M117.776815 . 28808052 PMC5633120

[24] Fedorov R, Böhl M, Tsiavaliaris G, Hartmann FK, Taft MH, Baruch P, et al The mechanism of pentabromopseudilin inhibition of myosin motor activity. Nat Struct Mol Biol. 2009;16(1):80–8. 10.1038/nsmb.1542 19122661

[25] Islam K, Chin HF, Olivares AO, Saunders LP, De La Cruz EM, Kapoor TM. A myosin V inhibitor based on privileged chemical scaffolds. Angew Chem Int Ed. 2010;49(45):8484–8. 10.1002/anie.201004026 20878825 PMC3063097

[26] Heissler SM, Selvadurai J, Bond LM, Fedorov R, Kendrick-Jones J, Buss F, et al Kinetic properties and small-molecule inhibition of human myosin-6. FEBS Lett. 2012;586(19):3208–14. 10.1016/j.febslet.2012.07.014 . 22884421 PMC3527664

[27] Wollenberg RD, Taft MH, Giese S, Thiel C, Balázs Z, Giese H, et al Phenamacril is a reversible and non-competitive inhibitor of *Fusarium* class I myosin. J Biol Chem. 2018. 10.1074/jbc.RA118.005408 PMC6349130 30504222

[28] Zhang C, Chen Y, Yin Y, Ji H, Shim W, Hou Y, et al A small molecule species specifically inhibits *Fusarium* myosin I. Environmental Microbiology. 2015;17(8):2735–46. 10.1111/1462-2920.12711 25404531

[29] Li H, Diao Y, Wang J, Chen C, Ni J, Zhou M. JS399-19, a new fungicide against wheat scab. Crop Protection. 2008;27(1):90–5. 10.1016/j.cropro.2007.04.010.

[30] Zheng Z, Zhang Y, Wu X, Yang H, Ma L, Zhou M. FoMyo5 motor domain substitutions (Val151 to Ala and Ser418 to Thr) cause natural resistance to fungicide phenamacril in *Fusarium oxysporum*. Pestic Biochem Physiol. 2018;147:119–26. Epub 2018/06/24. 10.1016/j.pestbp.2017.12.007 . 29933981

[31] Chen Y, Wang W, Zhang A, Gu C, Zhou M, Gao T. Activity of the fungicide JS399-19 against *Fusarium* head blight of wheat and the risk of resistance. Agricultural Sciences in China. 2011;10(12):1906–13. 10.1016/s1671-2927(11)60191-0

[32] Hou Y, Qu X, Mao X, Kuang J, Duan Y, Song X, et al Resistance mechanism of *Fusarium fujikuro*i to phenamacril in the field. Pest Management Science. 2018;74(3):607–16. 10.1002/ps.4742 28960890

[33] Li B, Zheng Z, Liu X, Cai Y, Mao X, Zhou M. Genotypes and characteristics of phenamacril-resistant mutants in *Fusarium asiaticum*. Plant Dis. 2016;100(8):1754–61. Epub 2016/08/01. 10.1094/PDIS-02-16-0169-RE . 30686221

[34] Lu Q, Li J, Ye F, Zhang M. Structure of myosin-1c tail bound to calmodulin provides insights into calcium-mediated conformational coupling. Nat Struct Mol Biol. 2015;22(1):81–8. Epub 2014/12/02. 10.1038/nsmb.2923 . 25437912

[35] Shen M, Zhang N, Zheng S, Zhang WB, Zhang HM, Lu Z, et al Calmodulin in complex with the first IQ motif of myosin-5a functions as an intact calcium sensor. Proc Natl Acad Sci USA. 2016;113(40):E5812–E20. Epub 2016/09/21. 10.1073/pnas.1607702113 . 27647889 PMC5056106

[36] Kollmar M, Durrwang U, Kliche W, Manstein DJ, Kull FJ. Crystal structure of the motor domain of a class-I myosin. Embo J. 2002;21(11):2517–25. Epub 2002/05/29. 10.1093/emboj/21.11.2517 . 12032065 PMC126035

[37] Allingham JS, Smith R, Rayment I. The structural basis of blebbistatin inhibition and specificity for myosin II. Nat Struct Mol Biol. 2005;12(4):378–9. Epub 2005/03/08. 10.1038/nsmb908 . 15750603

[38] Preller M, Chinthalapudi K, Martin R, Knölker H-J, Manstein DJ. Inhibition of myosin ATPase activity by halogenated pseudilins: a structure-activity study. J Med Chem. 2011;54(11):3675–85. 10.1021/jm200259f 21534527

[39] Winkelmann DA, Forgacs E, Miller MT, Stock AM. Structural basis for drug-induced allosteric changes to human β-cardiac myosin motor activity. Nat Commun. 2015;6:7974-. 10.1038/ncomms8974 . 26246073 PMC4918383

[40] Coureux PD, Sweeney HL, Houdusse A. Three myosin V structures delineate essential features of chemo-mechanical transduction. Embo J. 2004;23(23):4527–37. Epub 2004/10/29. 10.1038/sj.emboj.7600458 . 15510214 PMC533045

[41] Coureux PD, Wells AL, Ménétrey J, Yengo CM, Morris CA, Sweeney HL, et al A structural state of the myosin V motor without bound nucleotide. Nature. 2003;425(6956):419–23. 10.1038/nature01927 14508494

[42] Yang Y, Gourinath S, Kovács M, Nyitray L, Reutzel R, Himmel DM, et al Rigor-like structures from muscle myosins reveal key mechanical elements in the transduction pathways of this allosteric motor. Structure. 2007;15(5):553–64. 10.1016/j.str.2007.03.010 17502101

[43] Kovacs M, Toth J, Hetenyi C, Malnasi-Csizmadia A, Sellers JR. Mechanism of blebbistatin inhibition of myosin II. J Biol Chem. 2004;279(34):35557–63. Epub 2004/06/19. 10.1074/jbc.M405319200 . 15205456

[44] Ménétrey J, Isabet T, Ropars V, Mukherjea M, Pylypenko O, Liu X, et al Processive steps in the reverse direction require uncoupling of the lead head lever arm of myosin VI. Mol Cell. 2012;48(1):75–86. 10.1016/j.molcel.2012.07.034 22940248 PMC3472048

[45] Ménétrey J, Bahloul A, Wells AL, Yengo CM, Morris CA, Sweeney HL, et al The structure of the myosin VI motor reveals the mechanism of directionality reversal. Nature. 2005;435(7043):779–85. 10.1038/nature03592 15944696 PMC2762700

[46] Frye JJ, Klenchin VA, Bagshaw CR, Rayment I. Insights into the importance of hydrogen bonding in the γ-phosphate binding pocket of myosin: structural and functional studies of Serine 236. Biochemistry. 2010;49(23):4897–907. 10.1021/bi1001344 20459085 PMC2946171

[47] Kredics L, Narendran V, Shobana CS, Vagvolgyi C, Manikandan P, Indo-Hungarian Fungal Keratitis Working G. Filamentous fungal infections of the cornea: a global overview of epidemiology and drug sensitivity. Mycoses. 2015;58(4):243–60. Epub 2015/03/03. 10.1111/myc.12306 . 25728367

[48] Sun S, Lui Q, Han L, Ma Q, He S, Li X, et al Identification and characterization of *Fusarium proliferatum*, a new species of fungi that cause fungal keratitis. Sci Rep. 2018;8(1):4859 Epub 2018/03/22. 10.1038/s41598-018-23255-z . 29559666 PMC5861105

[49] Tarabishy AB, Aldabagh B, Sun Y, Imamura Y, Mukherjee PK, Lass JH, et al MyD88 regulation of *Fusarium keratitis* is dependent on TLR4 and IL-1R1 but not TLR2. J Immunol. 2008;181(1):593–600. Epub 2008/06/21. 10.4049/jimmunol.181.1.593 . 18566426 PMC3909484

[50] Mahmoudi S, Masoomi A, Ahmadikia K, Tabatabaei SA, Soleimani M, Rezaie S, et al Fungal keratitis: An overview of clinical and laboratory aspects. Mycoses. 2018;61(12):916–30. Epub 2018/07/12. 10.1111/myc.12822 . 29992633

[51] Wang L, Sun S, Jing Y, Han L, Zhang H, Yue J. Spectrum of fungal keratitis in central China. Clin Exp Ophthalmol. 2009;37(8):763–71. Epub 2009/11/03. 10.1111/j.1442-9071.2009.02155.x . 19878220

[52] Kabsch W. XDS. Acta Crystallogr D Biol Crystallogr. 2010;66(Pt 2):125–32. Epub 01/22. 10.1107/S0907444909047337 . 20124692 PMC2815665

[53] Münnich S, Taft MH, Manstein DJ. Crystal structure of human myosin 1c—the motor in GLUT4 exocytosis: implications for Ca^2+^ regulation and 14-3-3 binding. J Mol Biol 2014;426(10):2070–81. 10.1016/j.jmb.2014.03.004 24636949

[54] Nam Y, Orfali R, Liu T, Yu K, Cui M, Wulff H, et al Structural insights into the potency of SK channel positive modulators. Scientific Reports. 2017;7(1):17178 10.1038/s41598-017-16607-8 29214998 PMC5719431

[55] Emsley P, Lohkamp B, Scott WG, Cowtan K. Features and development of coot. Acta Crystallogr, Sect D: Biol Crystallogr. 2010;66(Pt 4):486–501. Epub 03/24. 10.1107/S0907444910007493 . 20383002 PMC2852313

[56] Adams PD, Afonine PV, Bunkóczi G, Chen VB, Davis IW, Echols N, et al PHENIX: a comprehensive Python-based system for macromolecular structure solution. Acta Crystallogr D Biol Crystallogr. 2010;66(Pt 2):213–21. Epub 01/22. 10.1107/S0907444909052925 . 20124702 PMC2815670

[57] GJ Kleywegt JZ, M Kjeldgaard & TA Jones. International tables for crystallography. 2001;F(17.1):353–6, 66–67.

[58] Yu J, Hamari Z, Han K, Seo J, Reyes-Domínguez Y, Scazzocchio C. Double-joint PCR: a PCR-based molecular tool for gene manipulations in filamentous fungi. Fungal Genet Biol. 2004;41(11):973–81. 10.1016/j.fgb.2004.08.001 15465386

[59] Zheng Z, Gao T, Zhang Y, Hou Y, Wang J, Zhou M. FgFim, a key protein regulating resistance to the fungicide JS399-19, asexual and sexual development, stress responses and virulence in *Fusarium graminearum*. Mol Plant Pathol. 2014;15(5):488–99. 10.1111/mpp.12108 24299032 PMC6638886

