# Supplementary figures and images for: Structural basis of Fusarium myosin I inhibition by phenamacril
Source: PLoS Pathog. 2020 Mar 12;16(3):e1008323. doi: 10.1371/journal.ppat.1008323 (PMC7100991; doi:10.1371/journal.ppat.1008323)

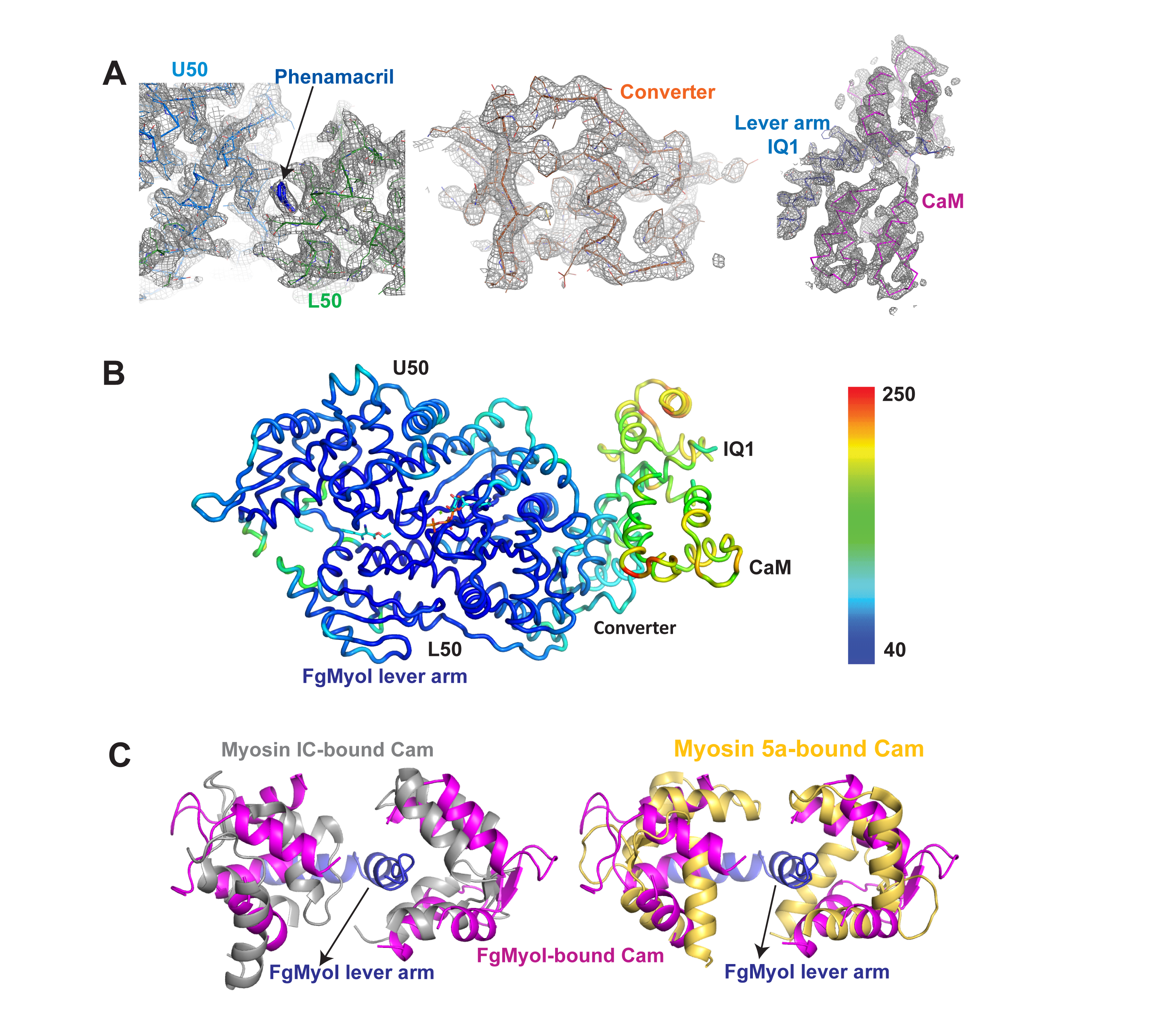

Supplement: S1 Fig — (A) FgMyoI-phenamacril-FgCaM complex structure with 2Fo-Fc map contoured at 1 σ for the myosin domains, and contoured at 0.7 σ for FgCaM. (B) Cartoon presentation of the FgMyoI-phenamacril-FgCaM complex structure with B factors of the structure depicted in a rainbow color code from blue for B factors around 40 Å2, to red for B factors about 250 Å2. (C) Structural comparison of FgMyoI-bound FgCaM with calmodulins that bind myosin Ic (left panel, PDB: 4R8G) and myosin 5a (right panel, PDB: 4ZLK), respectively. The RMSD between FgMyoI-bound FgCaM and myosin Ic-bound calmodulin, and myosin 5a-bound calmodulin are 6.1, and 6.9 Å, respectively. FgCaM and FgMyoI-bound FgCaM is colored in magenta; myosin Ic-bound calmodulin, in gray; and myosin 5a-bound CaM in yellow. Both lever arms of myosins Ic and 5a are omitted for clarity, except the IQ1 helix of FgMyoI, which is colored in blue. (TIF) [file ppat.1008323.s001.tif]

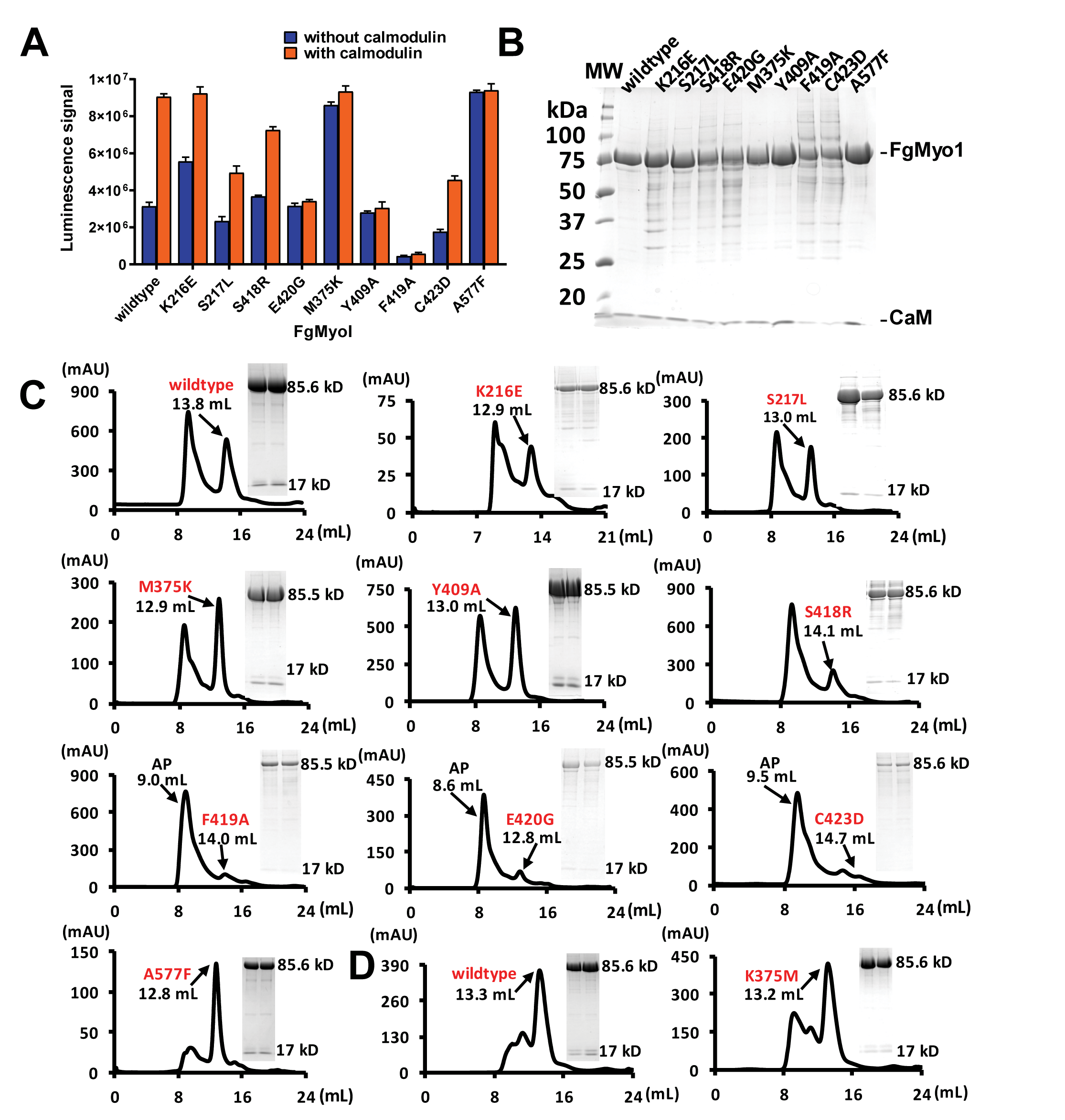

Supplement: S2 Fig — (A) ATPase activity (500 nM FgMyoI+/-100 nM CaM); n = 3, error bars = SD. (B) Protein purity (SDS PAGE). (C) Size exclusion chromatograms of wildtype and mutant FgMyoI. (D) Size exclusion chromatograms of wildtype and mutant MgMyoI. SDS PAGE inserts in the SEC panels show the proteins from the two top elution fractions. Note that FgMyoI F419A, E420G, and C423D are largely aggregated and co-purified calmodulin is not or only poorly visible. AP: Aggregation peak. (TIF) [file ppat.1008323.s002.tif]

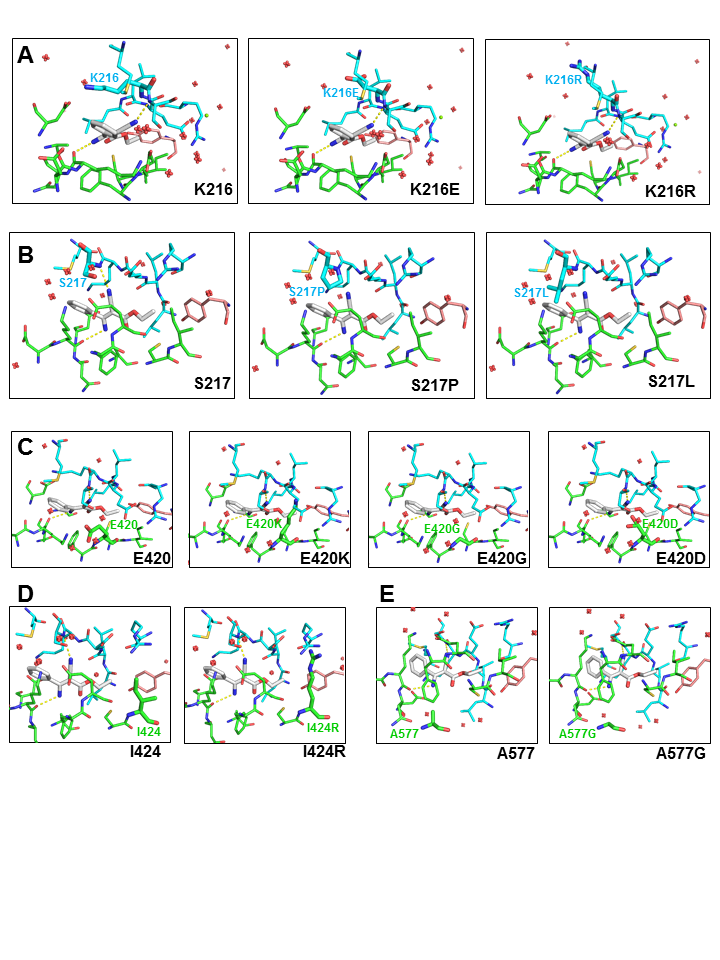

Supplement: S3 Fig — (A) K216 mutations. (B) S217 mutations. (C) E420 mutations. (D) I424 mutation. (E) A577 mutation. Resistance mutations were modeled in the phenamacril binding site with wildtype and mutant pockets shown in the same orientation next to each other. (TIF) [file ppat.1008323.s003.tif]

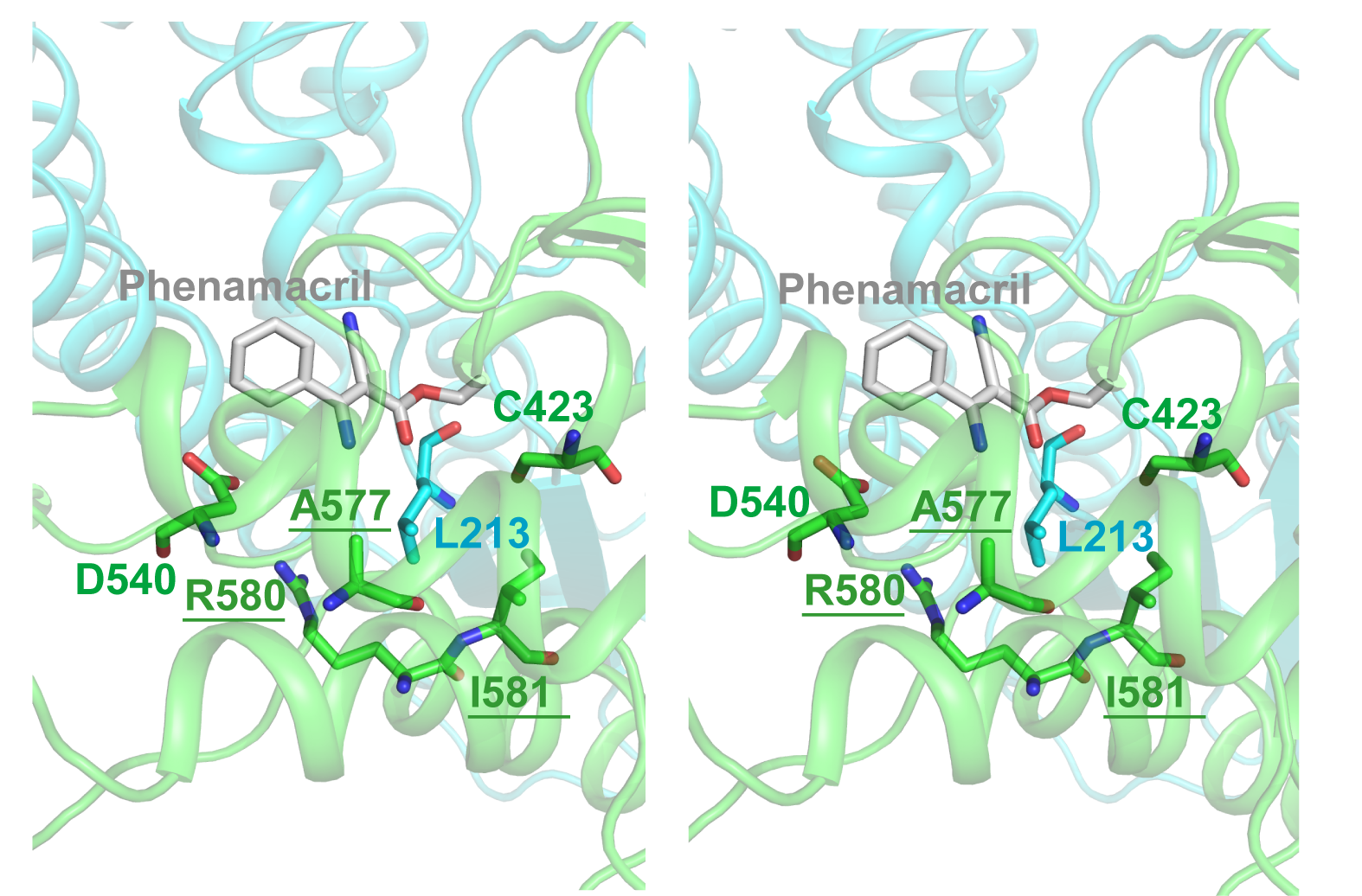

Supplement: S4 Fig — (TIF) [file ppat.1008323.s004.tif]

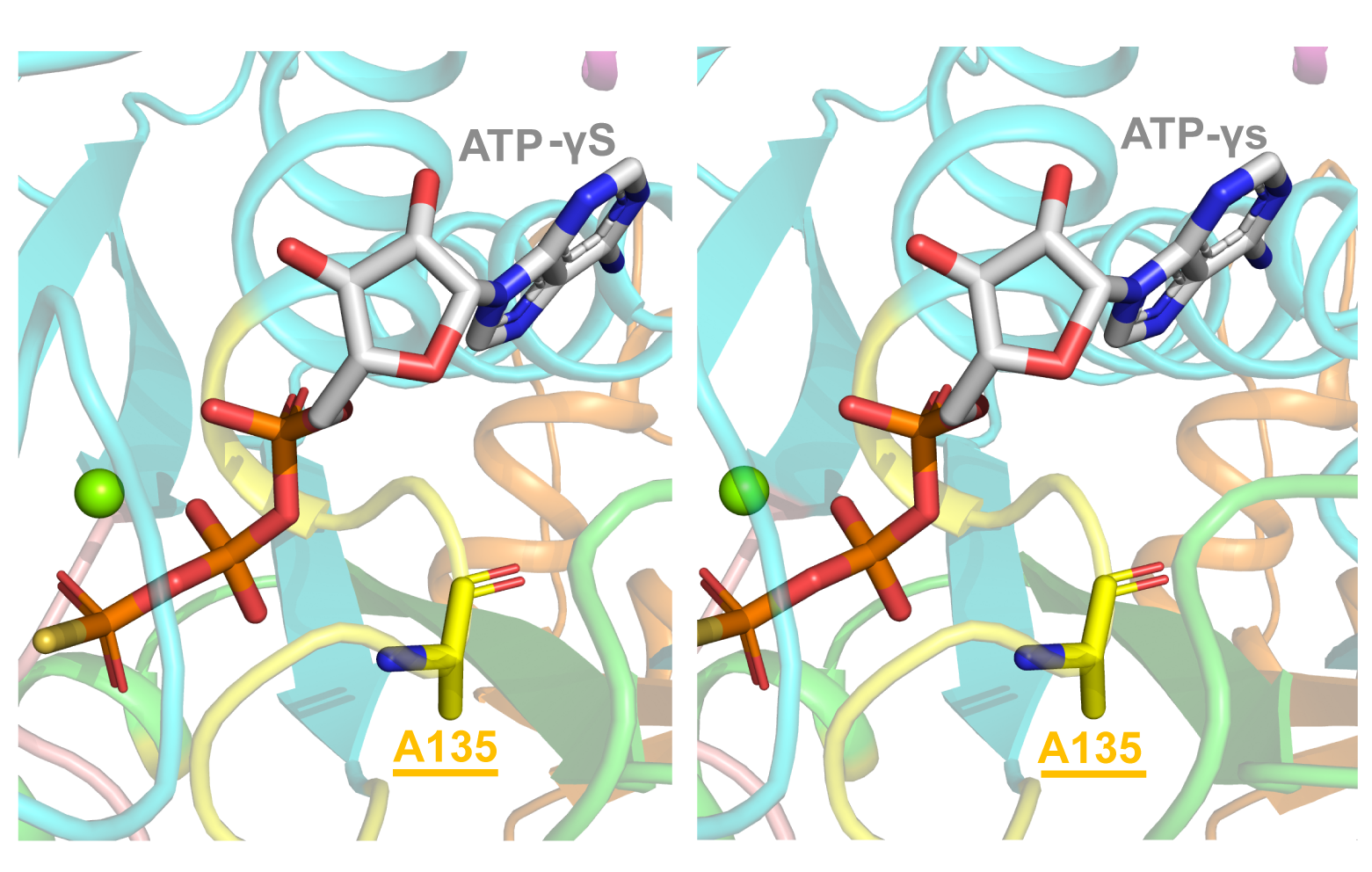

Supplement: S5 Fig — (TIF) [file ppat.1008323.s005.tif]

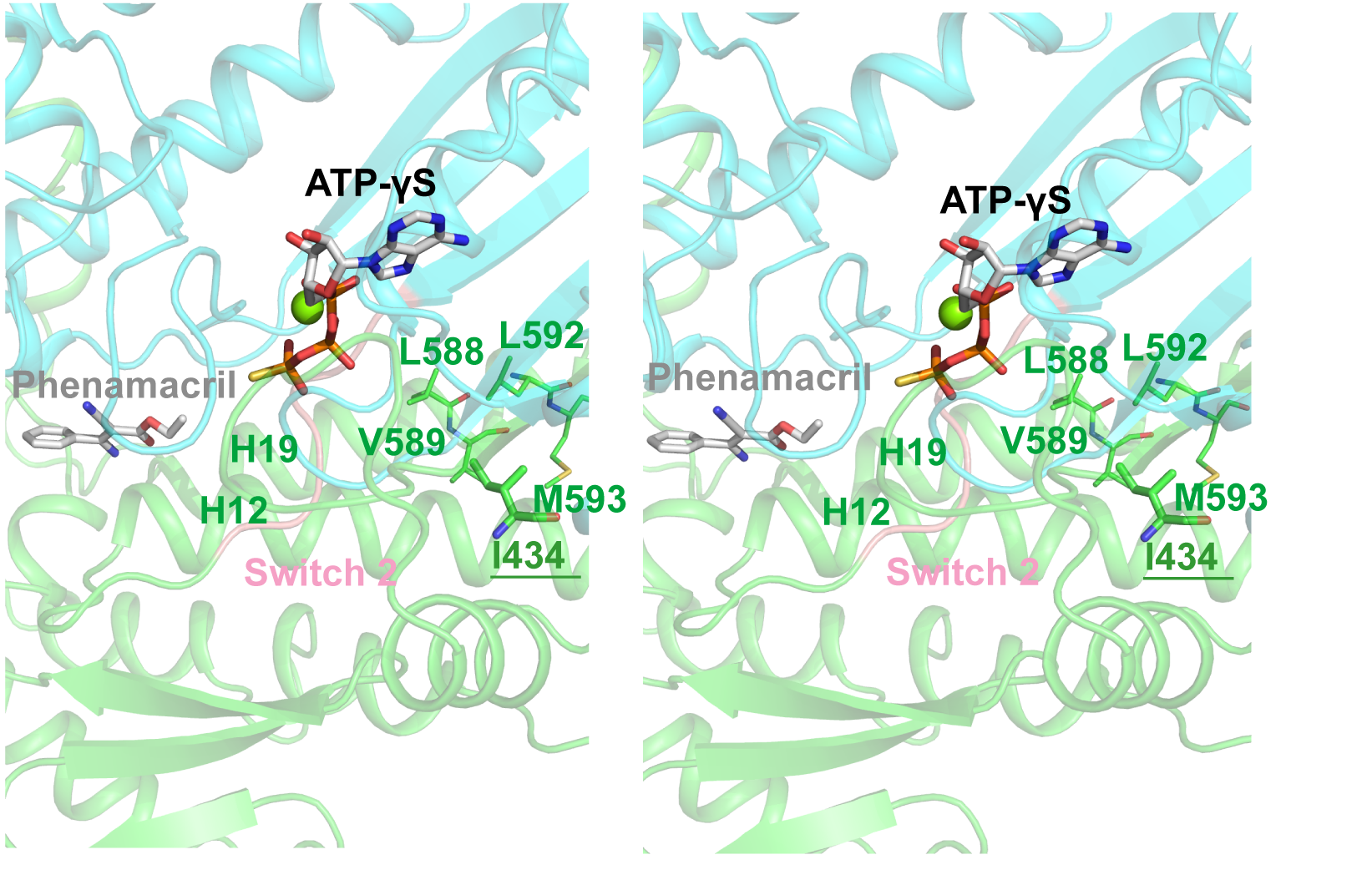

Supplement: S6 Fig — (TIF) [file ppat.1008323.s006.tif]

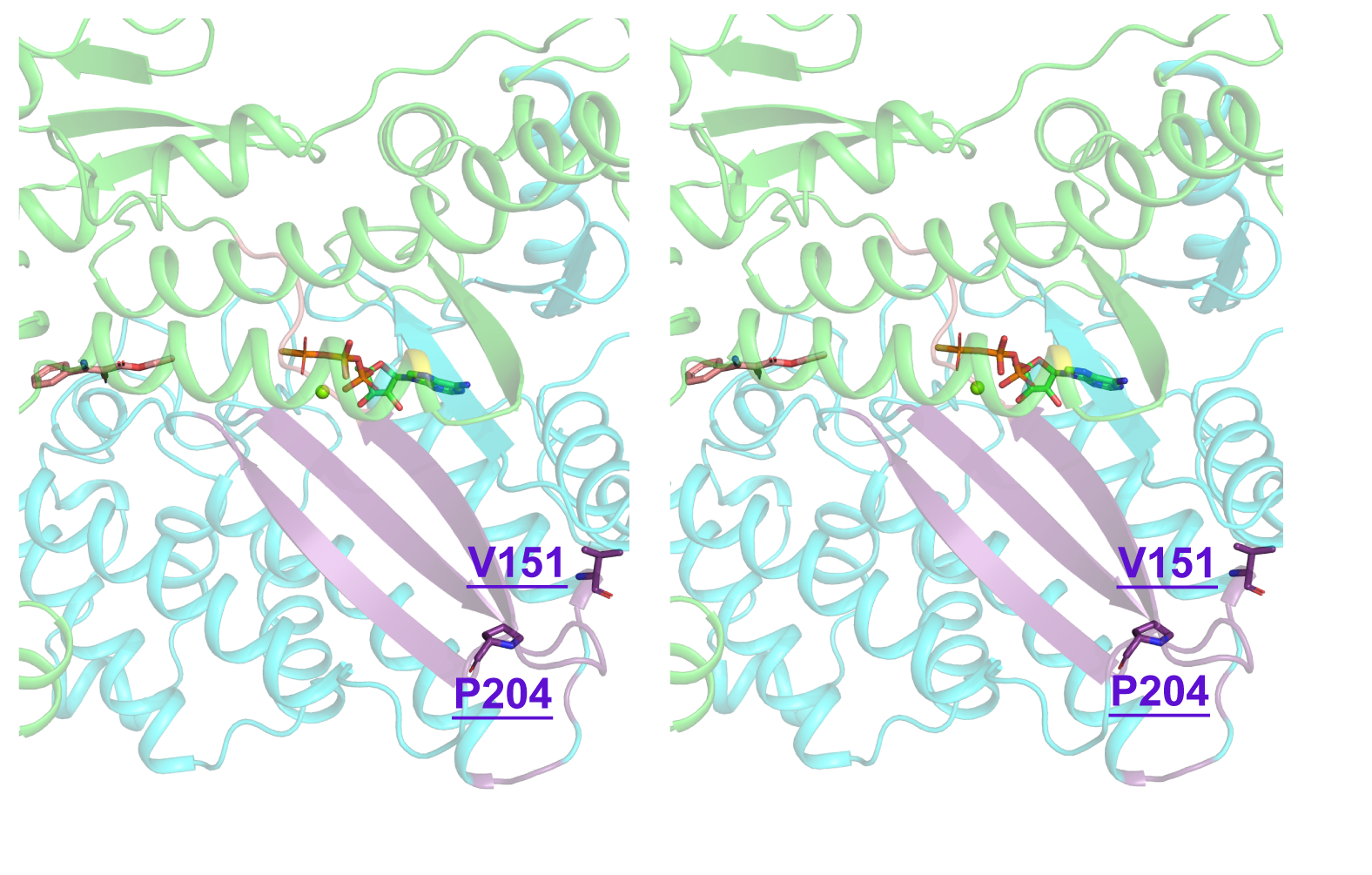

Supplement: S7 Fig — (TIF) [file ppat.1008323.s007.tif]
