# Supplementary material for: Structural basis of Fusarium myosin I inhibition by phenamacril
Source: PLoS Pathog. 2020 Mar 12;16(3):e1008323. doi: 10.1371/journal.ppat.1008323 (PMC7100991; doi:10.1371/journal.ppat.1008323)
Supplement: S1 Table — Dictyostelium discoideum class 1 myosin: PDB 1LKX; Rattus norvegicus class 1 myosin: PDB 5V7X; Argopecten irradians class 2 myosin: PDB 1DFL; Placopecten magellanicus class 2 myosin: PDB 2EC6; Doryteuthis pealeii class 2 myosin: PDB 3I5G; Bos Taurus class 2 myosin: PDB 5N6A; Dictyostelium discoideum class 2 myosin: PDB 2Y8I; Gallus gallus class 5 myosin: PDB 1OE9; Mus musculus class 5 myosin: PDB 4ZLK; Homo sapiens class 10 myosin: PDB 5KG8; Sus scrofa class 6 myosin: PDB 4ANJ. (PDF) [file ppat.1008323.s008.pdf]

S1 Table. Alignment of the FgMyoI (1-736) sequence with sequences of myosins with solved motor domain structures.

Dictyostelium discoideum class 1 myosin: PDB 1LKX; *Rattus norvegicus* class 1 myosin: PDB 5V7X; *Argopecten irradians* class 2 myosin: PDB 1DFL; *Placopecten magellanicus* class 2 myosin: PDB 2EC6; *Doryteuthis pealeii* class 2 myosin: PDB 3I5G; *Bos Taurus* class 2 myosin: PDB 5N6A; *Dictyostelium discoideum* class 2 myosin: PDB 2Y8I; *Gallus gallus* class 5 myosin: PDB 1OE9; *Mus musculus* class 5 myosin: PDB 4ZLK; *Homo sapiens* class 10 myosin: PDB 5KG8; *Sus scrofa* class 6 myosin: PDB 4ANJ.

```
                                                                                  1
FgMyoI_1-736              ........................................................MGIS
1LKX_Myo1_D.discoideum    ............................................................
5V7X_Myo1_R.norvegicus    ............................................................
1DFL_Myo2_A.irradians     ...............................FSDPDFQYLAVDRKKLMKEQTAAFDGKKN
2EC6_Myo2_P.magellanicus  ...........................MNIDFNDPDFQYLAVDRKKMMKEQTAPFDGKKN
3I5G_Myo2_D.pealeii       ...........................MTMDFSDPDMEFLCLTRQKLMEATSIPFDGKKN
5N6A_Myo2_B.taurus        ........................MVDAEMAAFGEAAPYLRKSEKERLEAQTRPFDLKKD
2Y8I_Myo2_D.discoideum    ..........................NPIHDRTSDYHKYLKVKQGDSDLFKLTVSDKRYI
1OE9_Myo5_G.gallus        ................................................MAASELYTKYAR
4ZLK_Myo5_M.musculus      MSYYHHHHHHDYKDDDDKNIPTTENLYFQGAMGIRNSKAYVDELTSPAMAASELYTKFAR
5KG8_Myo10_H.sapiens      ....................................................NFFTEGTR
4ANJ_Myo6_S.scrofa        ......................................................MEDGKP

                              10        20        30                              40
FgMyoI_1-736              RRPKNKGAGAAADGASGGAKPKKATFETTKK.......................KEIGVS
1LKX_Myo1_D.discoideum    ..........................MIPKT.......................KAEGVP
5V7X_Myo1_R.norvegicus    ...................MAKKEVKSSLLD.......................NMIGVG
1DFL_Myo2_A.irradians     CWVPDEKE.GFASAEIQSSKGDEITVKIVADSSTRTVKKDDIQS......MNPPKFEKLE
2EC6_Myo2_P.magellanicus  CWVPDPKE.GFASAEIQSSKGEEITVKIVSDNSTRTVKKDDIQQ......MNPPKFEKLE
3I5G_Myo2_D.pealeii       CWVPDPDF.GFVGAEIQSTKGDEVTVKTDKTQETRVVKKDDIGQ......RNPPKFEMNM
5N6A_Myo2_B.taurus        VFVPDDKE.EFVKATILSREGGKVTAETEHG.KTVTVKEDQVLQ......QNPPKFDKIE
2Y8I_Myo2_D.discoideum    WYNPDPKERDSYECGEIVSETSDSFTFKTVDGQDRQVKKDDANQ......RNPIKFDGVE
1OE9_Myo5_G.gallus        VWIPDPEEVWKSAELLKDYKPGDKVLQLRLEEGKDLEYCLDPKTKELPPLRNPDILVGEN
4ZLK_Myo5_M.musculus      VWIPDPEEVWKSAELLKDYKPGDKVLLLHLEEGKDLEYRLDPKTGELPHLRNPDILVGEN
5KG8_Myo10_H.sapiens      VWLRENGQ....HFPSTVNSCAEGIVVFRTDYGQVFTYKQSTITHQKVTAMHPTNEEGVD
4ANJ_Myo6_S.scrofa        VWAPHPTDGFQVGNIVDIGPDSLTIEPLNQKGKTFLALINQVFP......AEEDSKKDVE

                                 50        60         70        80         90
FgMyoI_1-736              DLTLLSKVSNEAINENLKKRFEG.REIYTYIGHVLVSVNPFRDLG.IYTDDVLQSYMGKN
1LKX_Myo1_D.discoideum    DFVLLNQITENAFIENLTMRHKS.DNIYTYIGDVVISTNPFKNLN.IYKESDIKAYNGRY
5V7X_Myo1_R.norvegicus    DTVLLEPLNEETFIDNLKKRFDH.NEIYTYIGSVVISVNPYRSLP.IYSPEKVEDYRNRN
1DFL_Myo2_A.irradians     DMANMTYLNEASVLYNLRSRYTS.GLIYTYSGLFCIAVNPYRRLP.IYTDSVIAKYRGKR
2EC6_Myo2_P.magellanicus  DMANMTYLNEASVLNNLRGRYTA.GLIYTYSGLFCIAVNPYRRLP.IYTDSVIAKYRGKR
3I5G_Myo2_D.pealeii       DMANLTFLNEASILHNLRSRYES.GFIYTYSGLFCIAINPYRRLP.IYTQGLVDKYRGKR
5N6A_Myo2_B.taurus        DMAMLTFLHEPAVLYNLKERYAS.WMIYTYSGLFCVTINPYKWLP.VYNAEVVAAYRGKK
2Y8I_Myo2_D.discoideum    DMSELSYLNEPAVFHNLRVRYNQ.DLIYTYSGLFLVAVNPFKRIP.IYTQEMVDIFKGRR
1OE9_Myo5_G.gallus        DLTALSYLHEPAVLHNLKVRFIDSKLIYTYCGIVLVAINPYEQLP.IYGEDIINAYSGQN
4ZLK_Myo5_M.musculus      DLTALSYLHEPAVLHNLRVRFIDSKLIYTYCGIVLVAINPYEQLP.IYGEDIINAYSGQN
5KG8_Myo10_H.sapiens      DMASLTELHGGSIMYNLFQRYKR.NQIYTYIGSILASVNPYQPIAGLYEPATMEQYSRRH
4ANJ_Myo6_S.scrofa        DNCSLMYLNEATLLHNIKVRYSK.DRIYTYVANILIAVNPYFDIPKIYSSETIKSYQGKS

                        100       110       120       130       140       150
FgMyoI_1-736              RLEMPPHVFAIAEASYYNMKAYSDNQCVIISGESGAGKTEAAKRIMQYIASVSGGESGDI
1LKX_Myo1_D.discoideum    KYEMPPHMYALANDAYRSMRQSQENQCVIISGESGAGKTEASKKIMQFLTFVSSNQSPNG
5V7X_Myo1_R.norvegicus    FYELSPHIFALSDEAYRSLRDQDKDQCILITGESGAGKTEASKLVMSYVAAVCG.KGAEV
1DFL_Myo2_A.irradians     KTEIPPHLFSVADNAYQNMVTDRENQSCLITGESGAGKTENTKKVIMYLAKVACAVK...
2EC6_Myo2_P.magellanicus  KTEIPPHLFSVADNAYQNMVTDRENQSCLITGESGAGKTENTKKVIMYLAKVACAVK...
3I5G_Myo2_D.pealeii       RAEMPPHLFSIADNAYQYMLQDRENQSMLITGESGAGKTENTKKVIQYFALVAASLAGKK
5N6A_Myo2_B.taurus        RSEAPPHIFSISDNAYQYMLTDRENQSILITGESGAGKTVNTKRVIQYFAVIAAIGD...
2Y8I_Myo2_D.discoideum    RNEVAPHIFAISDVAYRSMLDDRQNQSLLITGESGAGKTENTKKVIQYLASVAGRNQ...
1OE9_Myo5_G.gallus        MGDMDPHIFAVAEEAYKQMARDERNQSIIVSGESGAGKTVSAKYAMRYFATVSGSAS...
4ZLK_Myo5_M.musculus      MGDMDPHIFAVAEEAYKQMARDERNQSIIVSGESGAGKTVSAKYAMRYFATVSGSAS...
5KG8_Myo10_H.sapiens      LGELPPHIFAIANECYRCLWKRHDNQCILISGESGAGKTESTKLILKFLSVISQQSL...
4ANJ_Myo6_S.scrofa        LGTMPPHVFAIADKAFRDMKVLKLSQSIIVSGESGAGKTENTKFVLRYLTESYGTGQ...

                                     160       170       180       190       200
FgMyoI_1-736              .............KQIKDMVLATNPLLESFGNAKTLRNNNSSRFGKYLQIYFNTQGEPVG
1LKX_Myo1_D.discoideum    .............ERISKMLLDSNPLLEAFGNAKTLRNDNSSRFGKYMEMQFNAVGSPIG
5V7X_Myo1_R.norvegicus    .............NQVKEQLLQSTPVLEAFGNAKTVRNDNSSRFGKYMDIEFDFKGDPLG
1DFL_Myo2_A.irradians     KKD..EEASDKKEGSLEDQIIQANPVLEAYGNAKTTRNNNSSRFGKFIRIHFGPTGKIAG
2EC6_Myo2_P.magellanicus  KKTSEEEEADQKKGSLEDQIIQANPVLEAYGNAKTTRNNNSSRFGKFIRIHFGPTGKIAG
3I5G_Myo2_D.pealeii       DKKEEEKKKDEKKGTLEDQIVQCNPVLEAYGNAKTTRNNNSSRFGKFIRIHFGTQGKIAG
5N6A_Myo2_B.taurus        ...RSKKEQATGKGTLEDQIIQANPALEAFGNAKTVRNDNSSRFGKFIRIHFGATGKLAS
2Y8I_Myo2_D.discoideum    .........ANGSGVLEQQILQANPILEAFGNAKTTRNNNSSRFGKFIEIQFNSAGFISG
1OE9_Myo5_G.gallus        ............EANVEEKVLASNPIMESIGNAKTTRNDNSSRFGKYIEIGFDKRYRIIG
4ZLK_Myo5_M.musculus      ............EANVEEKVLASNPIMESIGNAKTTRNDNSSRFGKYIEIGFDKRYRIIG
5KG8_Myo10_H.sapiens      .....ELSLKEKTSCVERAILESSPIMEAFGNAKTVYNNNSSRFGKFVQLNICQKGNIQG
4ANJ_Myo6_S.scrofa        ..............DIDDRIVEANPLLEAFGNAKTVRNNNSSRFGKFVEIHFNEKSSVVG
                                                                    ↑
                                                                   R189
```

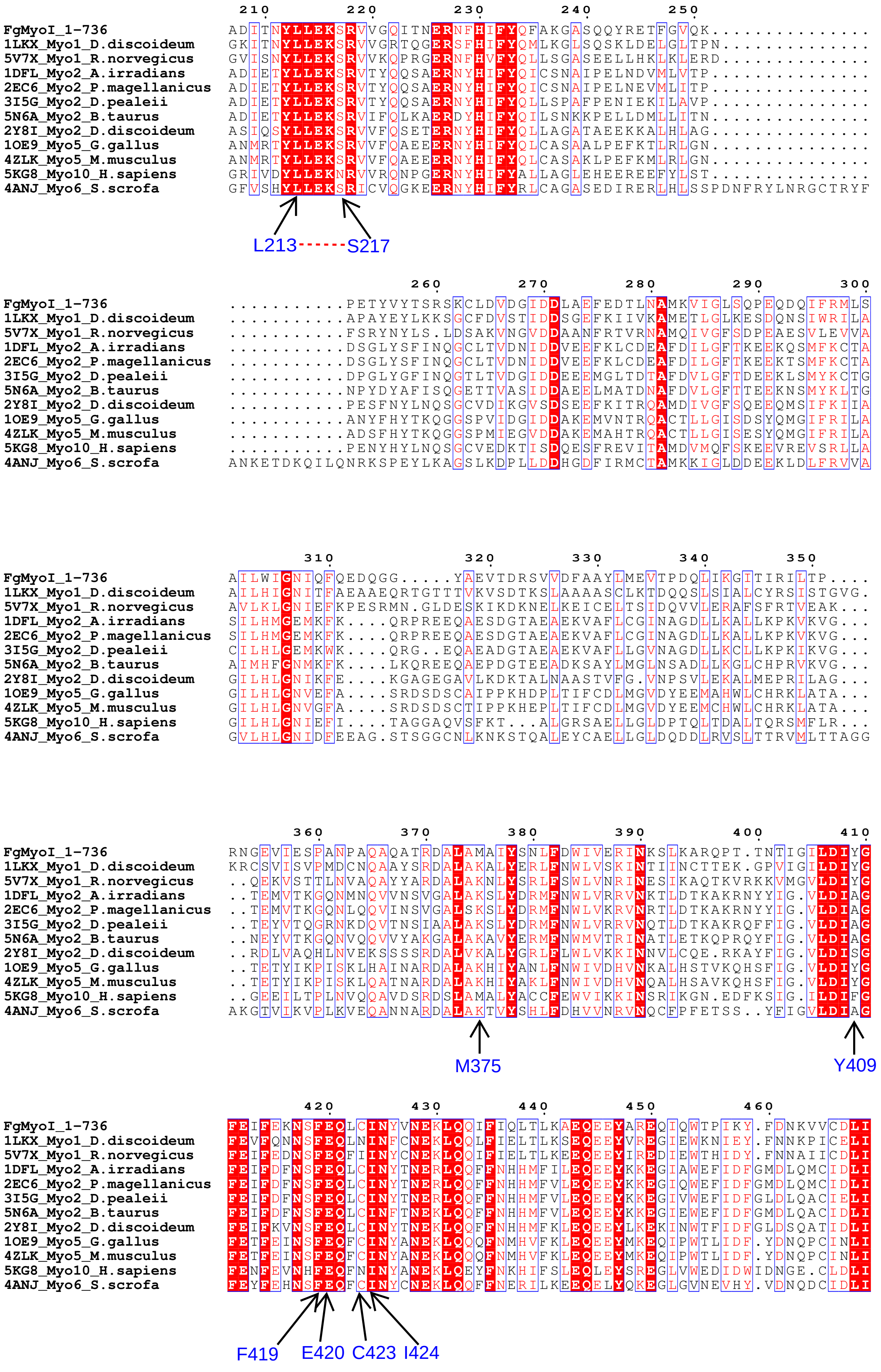
210 220 230 240 250
FgMyoI_1-736 ADITNYLLEKSRVVGQITNERNFHIFYQFAKGASQQYRETFGVQK...............
1LKX_Myo1_D.discoideum GKITNYLLEKSRVVGRTQGERSFHIFYQMLKGLSQSKLDELGLTPN..............
5V7X_Myo1_R.norvegicus GVISNYLLEKSRVVKQPRGERNFHVFYQLLSGASEELLHKLKLERD..............
1DFL_Myo2_A.irradians ADIETYLLEKSRVTYQQSAERNYHIFYQICSNAIPELNDVMLVTP...............
2EC6_Myo2_P.magellanicus ADIETYLLEKSRVTYQQSAERNYHIFYQICSNAIPELNEVMLITP...............
3I5G_Myo2_D.pealeii ADIETYLLEKSRVTYQQSAERNYHIFYQLLSPAFPENIEKILAVP...............
5N6A_Myo2_B.taurus ADIETYLLEKSRVIFQLKAERDYHIFYQILSNKKPELLDMLLITN...............
2Y8I_Myo2_D.discoideum ASIQSYLLEKSRVVFQSETERNYHIFYQLLAGATAEEKKALHLAG...............
1OE9_Myo5_G.gallus ANMRTYLLEKSRVVFQAEEERNYHIFYQLCASAALPEFKTLRLGN...............
4ZLK_Myo5_M.musculus ANMRTYLLEKSRVVFQAEEERNYHIFYQLCASAKLPEFKMLRLGN...............
5KG8_Myo10_H.sapiens GRIVDYLLEKNRVVRQNPGERNYHIFYALLAGLEHEEREEFYLST...............
4ANJ_Myo6_S.scrofa GFVSHYLLEKSRICVQGKEERNYHIFYRLCAGASEDIRERLHLSSPDNFRYLNRGCTRYF
L213 S217
260 270 280 290 300
FgMyoI_1-736 ...........PETYVYTSRSKCLDVDGIDDLAEEFEDTLNAMKVIGLSQPEQDQIFRMLS
1LKX_Myo1_D.discoideum ...........APAYEYLKKSGCFDVSTIDDSGEFKIIVKAMETLGLKESDQNSIWRILA
5V7X_Myo1_R.norvegicus ....FSRYNYLS.LDSAKVNGVDDAANFRTVRNAMQIVGFSDPEAESVLEVVA
1DFL_Myo2_A.irradians ...........DSGLYSFINQGCLTVDNIDDVEEFKLCDEAFDILGFTKEEKQSMFKCTA
2EC6_Myo2_P.magellanicus ...........DSGLYSFINQGCLTVDNIDDVEEFKLCDEAFDILGFTKEEKTSMFKCTA
3I5G_Myo2_D.pealeii ...........DPGLYGFINQGTLTVDGIDDEEEMGLTDTAFDVLGFTDEEKLSMYKCTG
5N6A_Myo2_B.taurus ...........NPYDYAFISQGETTVASIDDAEELMATDNAFDVLGFTTEEKNSMYKLTG
2Y8I_Myo2_D.discoideum ...........PESFNYLNQSGCVDIKGVSDSEEFKITRQAMDIVGFSQEEQMSIFKIIA
1OE9_Myo5_G.gallus ...........ANYFHYTKQGGSPVIDGIDDAKEMVNTRQACTLLGISDSYQMGIFRILA
4ZLK_Myo5_M.musculus ...........ADSFHYTKQGGSPMIEGVDDAKEMAHTRQACTLLGISESYQMGIFRILA
5KG8_Myo10_H.sapiens ...........PENYHYLNQSGCVEDKTISDQESFREVITAMDVMQFSKEEVREVSRLLA
4ANJ_Myo6_S.scrofa ANKETDKQILQNRKSPEYLKAGSLKDPLLDDHGDFIRMCTAMKKIGLDDEEKLDLFRVVA
310 320 330 340 350
FgMyoI_1-736 AILWIGNIQFQEDQGG.....YAEVTDRSVVDFAAYLMEVTPDQLIKGITIRILTP....
1LKX_Myo1_D.discoideum AILHIGNITFAEAAEQRTGTTTVKVSDTKSLAAAASCLKTDQQSLSIALCYRSISTGVG.
5V7X_Myo1_R.norvegicus AVLKLGNIEFKPESRMN.GLDESKIKDKNELKEICELTSIDQVVLERAFSFRTVEAK...
1DFL_Myo2_A.irradians SILHMGEMKFK....QRPREEQAESDGTAEAEKVAFLCGINAGDLLKALLKPKVKVG...
2EC6_Myo2_P.magellanicus SILHMGEMKFK....QRPREEQAESDGTAEAEKVAFLCGINAGDLLKALLKPKVKVG...
3I5G_Myo2_D.pealeii CILHLGEMKWK....QRG..EQAEADGTAEAEKVAFLLGVNAGDLLKCLLKPKIKVG...
5N6A_Myo2_B.taurus AIMHFGNMKFK....LKQREEQAEPDGTEEADKSAYLMGLNSADLLKGLCHPRVKVG...
2Y8I_Myo2_D.discoideum GILHLGNIKFE....KGAGEGAVLKDKTALNAASTVFG.VNPSVLEKALMEPRILAG...
1OE9_Myo5_G.gallus GILHLGNVEFA....SRDSDSCAIPPKHDPLTIFCDLMGVDYEEMAHWLCHRKLATA...
4ZLK_Myo5_M.musculus GILHLGNVGFA....SRDSDSCTIPPKHEPLTIFCDLMGVDYEEMCHWLCHRKLATA...
5KG8_Myo10_H.sapiens GILHLGNIEFI....TAGGAQVSFKT...ALGRSAELLGLDPTQLTDALTQRSMFLR...
4ANJ_Myo6_S.scrofa GVLHLGNIDFEEAG.STSGGCNLKNKSTQALEYCAELLGLDQDDLRVSLTTRVMLTTAGG
360 370 380 390 400 410
FgMyoI_1-736 RNGEVIESPANPAQAQATRDALAMAIYSNLFDWIVERINKSLKARQPT.TNTIGILDIYG
1LKX_Myo1_D.discoideum KRCSVISVPMDCNQAAYSRDALAKALYERLFNWLVSKINTIINCTTEK.GPVIGILDIYG
5V7X_Myo1_R.norvegicus ..QEKVSTTLNVAQAYYARDALAKNLYSRLFSWLVNRINESIKAQTKVRKKVMGVLDIYG
1DFL_Myo2_A.irradians ..TEMVTKGQNMNQVVNSVGALAKSLYDRMFNWLVRRVNKTLDTKAKRNYYIG.VLDIAG
2EC6_Myo2_P.magellanicus ..TEMVTKGQNLNQVINSVGALSKSLYDRMFNWLVKRVNRTLDTKAKRNYYIG.VLDIAG
3I5G_Myo2_D.pealeii ..TEYVTQGRNKDQVTNSIAALAKSLYDRMFNWLVRRVNQTLDTKAKRQFFIG.VLDIAG
5N6A_Myo2_B.taurus ..NEYVTKGQNVQQVYYAKGALAKAVYERMFNWMVTRINATLETKQPRQYFIG.VLDIAG
2Y8I_Myo2_D.discoideum ..RDLVAQHLNVEKSSSSRDALVKALYGRLFLWLVKKINNVLCQE.RKAYFIG.VLDISG
1OE9_Myo5_G.gallus ..TETYIKPISKLHAINARDALAKHIYANLFNWIVDHVNKALHSTVKQHSFIG.VLDIYG
4ZLK_Myo5_M.musculus ..TETYIKPISKLQATNARDALAKHIYAKLFNWIVDHVNQALHSAVKQHSFIG.VLDIYG
5KG8_Myo10_H.sapiens ..GEEILTPLNVQQAVDSRDSLAMALYACCFEWVIKKINSRIKGN.EDFKSIG.ILDIFG
4ANJ_Myo6_S.scrofa AKGTVIKVPLKVEQANNARDALAKTVYSHLFDHVVNRVNQCFPFETSS..YFIGVLDIAG
M375 Y409
420 430 440 450 460
FgMyoI_1-736 FEIFEKNSFEQLCINYVNEKLQQIFIQLTLKAEQEEYAREQIQWTPIKY.FDNKVVCDLI
1LKX_Myo1_D.discoideum FEVFQNNSFEQLNINFCNEKLQQLFIELTLKSEQEEYVREGIEWKNIEY.FNNKPICELI
5V7X_Myo1_R.norvegicus FEIFEDNSFEQFIINYCNEKLQQIFIELTLKEEQEEYIREDIEWTHIDY.FNNAIICDLI
1DFL_Myo2_A.irradians FEIFDFNSFEQLCINYTNERLQQFFNHHMFILEQEEYKKEGIAWEFIDFGMDLQMCIDLI
2EC6_Myo2_P.magellanicus FEIFDFNSFEQLCINYTNERLQQFFNHHMFVLEQEEYKKEGIQWEFIDFGMDLQMCIDLI
3I5G_Myo2_D.pealeii FEIFDFNSFEQLCINFTNEKLQQFFNHHMFVLEQEEYKKEGIVWEFIDFGLDLQACIELI
5N6A_Myo2_B.taurus FEIFDFNSFEQLCINYTNEKLQQFFNHHMFVLEQEEYKKEGIEWEFIDFGMDLQACIDLI
2Y8I_Myo2_D.discoideum FEIFKVNSFEQLCINYTNEKLQQFFNHHMFKLEQEEYLKEKINWTFIDFGLDSQATIDLI
1OE9_Myo5_G.gallus FETFEINSFEQFCINYANEKLQQQFNMHVFKLEQEEYMKEQIPWTLIDF.YDNQPCINLI
4ZLK_Myo5_M.musculus FETFEINSFEQFCINYANEKLQQQFNMHVFKLEQEEYMKEQIPWTLIDF.YDNQPCINLI
5KG8_Myo10_H.sapiens FENFEVNHFEQFNINYANEKLQEYFNKHIFSLEQLEYSREGLVWEDIDWIDNGE.CLDLI
4ANJ_Myo6_S.scrofa FEYFEHNSFEQFCINYCNEKLQQFFNERILKEEQELYQKEGLGVNEVHY.VDNQDCIDLI
F419 E420 C423 I424

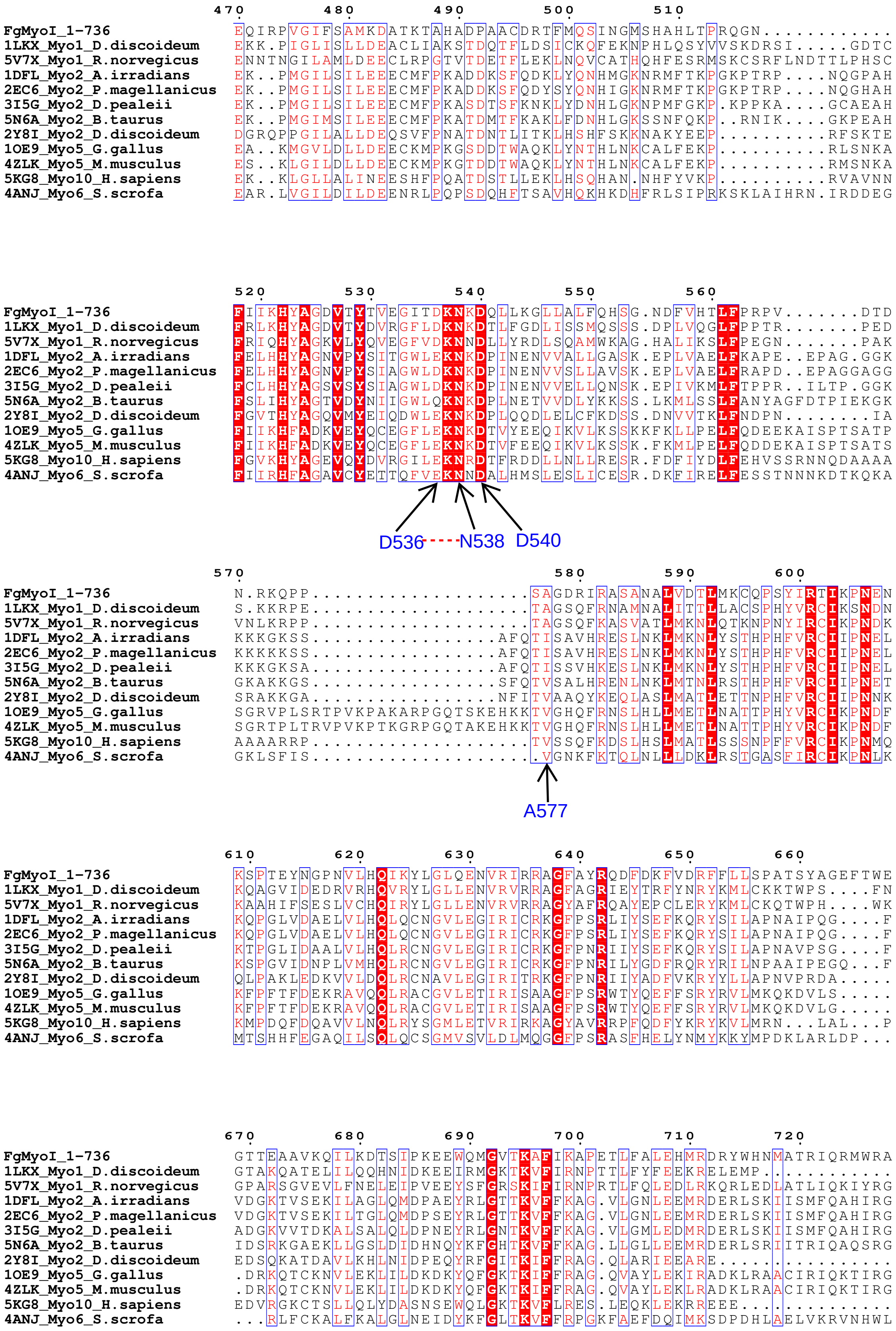
470 480 490 500 510
FgMyoI_1-736 EQIRPVGIFSAMKDATKTAHADPAACDRTFMQSINGMSHAHLTPRQGN............
1LKX_Myo1_D.discoideum EKK.PIGLISLLDEACLIAKSTDQTFLDSICKQFEKNPHLQSYVVSKDRSI.....GDTC
5V7X_Myo1_R.norvegicus ENNTNGILAMLDEECLRPGTVTDETFLEKLNQVCATHQHFESRMSKCSRFLNDTTLPHSC
1DFL_Myo2_A.irradians EK..PMGILSILEEECMFPKADDKSFQDKLYQNHMGKNRMFTKPGKPTRP....NQGPAH
2EC6_Myo2_P.magellanicus EK..PMGILSILEEECMFPKADDKSFQDYSYQNHIGKNRMFTKPGKPTRP....NQGHAH
3I5G_Myo2_D.pealeii EK..PMGILSILEEECMFPKASDTSFKNKLYDNHLGKNPMFGKP.KPPKA....GCAEAH
5N6A_Myo2_B.taurus EK..PMGIMSILEEECMFPKATDMTFKAKLFDNHLGKSSNFQKP..RNIK....GKPEAH
2Y8I_Myo2_D.discoideum DGRQPPGILALLDEQSVFPNATDNTLITKLHSHFSKKNAKYEEP..........RFSKTE
1OE9_Myo5_G.gallus EA..KMGVLDLLDEECKMPKGSDDTWAQKLYNTHLNKCALFEKP..........RLSNKA
4ZLK_Myo5_M.musculus ES..KLGILDLLDEECKMPKGTDDTWAQKLYNTHLNKCALFEKP..........RMSNKA
5KG8_Myo10_H.sapiens EK..KLGLLALINEESHFPQATDSTLLEKLHSQHAN.NHFYVKP..........RVAVNN
4ANJ_Myo6_S.scrofa EAR.LVGILDILDEENRLPQPSDQHFTSAVHQKHKDHFRLSIPRKSKLAIHRN.IRDDEG
520 530 540 550 560
FgMyoI_1-736 FIIKHYAGDVTYTVEGITDKNKDQLLKGLLALFQHSG.NDFVHTLFPRPV.......DTD
1LKX_Myo1_D.discoideum FRLKHYAGDVTYDVRGFLDKNKDTLFGDLISSMQSSS.DPLVQGLFPPTR.......PED
5V7X_Myo1_R.norvegicus FRIQHYAGKVLYQVEGFVDKNNDLLYRDLSQAMWKAG.HALIKSLFPEGN.......PAK
1DFL_Myo2_A.irradians FELHHYAGNVPYSITGWLEKNKDPINENVVALLGASK.EPLVAELFKAPE..EPAG.GGK
2EC6_Myo2_P.magellanicus FELHHYAGNVPYSIAGWLDKNKDPINENVVSLLAVSK.EPLVAELFRAPD..EPAGGAGG
3I5G_Myo2_D.pealeii FCLHHYAGSVSYSIAGWLDKNKDPINENVVELLQNSK.EPIVKMLFTPPR..ILTP.GGK
5N6A_Myo2_B.taurus FSLIHYAGTVDYNIIGWLQKNKDPLNETVVDLYKKSS.LKMLSSLFANYAGFDTPIEKGK
2Y8I_Myo2_D.discoideum FGVTHYAGQVMYEIQDWLEKNKDPLQQDLELCFKDSS.DNVVTKLFNDPN........IA
1OE9_Myo5_G.gallus FIIKHFADKVEYQCEGFLEKNKDTVYEEQIKVLKSSKKFKLLPELFQDEEKAISPTSATP
4ZLK_Myo5_M.musculus FIIKHFADKVEYQCEGFLEKNKDTVFEEQIKVLKSSK.FKMLPELFQDDEKAISPTSATS
5KG8_Myo10_H.sapiens FGVKHYAGEVQYDVRGILEKNRDTFRDDLLNLLRESR.FDFIYDLFEHVSSRNNQDAAAA
4ANJ_Myo6_S.scrofa FIIRHFAGAVCYETTQFVEKNNDALHMSLESLICESR.DKFIRELFESSTNNNKDTKQKA
D536
N538
D540
570 580 590 600
FgMyoI_1-736 N.RKQPP....................SAGDRIRASANALVDTLMKCQPSYIRTIKPNEN
1LKX_Myo1_D.discoideum S.KKRPE....................TAGSQFRNAMNALITTLLACSPHYVRCIKSNDN
5V7X_Myo1_R.norvegicus VNLKRPP....................TAGSQFKASVATLMKNLQTKNPNYIRCIKPNDK
1DFL_Myo2_A.irradians KKKGKSS.................AFQTISAVHRESLNKLMKNLYSTHPHFVRCIIPNEL
2EC6_Myo2_P.magellanicus KKKKKSS.................AFQTISAVHRESLNKLMKNLYSTHPHFVRCIIPNEL
3I5G_Myo2_D.pealeii KKKGKSA.................AFQTISSVHKESLNKLMKNLYSTHPHFVRCIIPNEL
5N6A_Myo2_B.taurus GKAKKGS.................SFQTVSALHRENLNKLMTNLRSTHPHFVRCIIPNET
2Y8I_Myo2_D.discoideum SRAKKGA.................NFITVAAQYKEQLASLMATLETTNPHFVRCIIPNNK
1OE9_Myo5_G.gallus SGRVPLSRTPVKPAKARPGQTSKEHKKTVGHQFRNSLHLLMETLNATTPHYVRCIKPNDF
4ZLK_Myo5_M.musculus SGRTPLTRVPVKPTKGRPGQTAKEHKKTVGHQFRNSLHLLMETLNATTPHYVRCIKPNDF
5KG8_Myo10_H.sapiens AAAARRP....................TVSSQFKDSLHSLMATLSSSNPFFVRCIKPNMQ
4ANJ_Myo6_S.scrofa GKLSFIS.....................VGNKFKTQLNLLLDKLRSTGASFIRCIKPNLK
A577
610 620 630 640 650 660
FgMyoI_1-736 KSPTEYNGPNVLHQIKYLGLQENVRIRRAGFAYRQDFDKFVDRFFLLSPATSYAGEFTWE
1LKX_Myo1_D.discoideum KQAGVIDEDRVRHQVRYLGLLENVRVRRAGFAGRIEYTRFYNRYKMLCKKTWPS....FN
5V7X_Myo1_R.norvegicus KAAHIFSESLVCHQIRYLGLLENVRVRRAGYAFRQAYEPCLERYKMLCKQTWPH....WK
1DFL_Myo2_A.irradians KQPGLVDAELVLHQLQCNGVLEGIRICRKGFPSRLIYSEFKQRYSILAPNAIPQG....F
2EC6_Myo2_P.magellanicus KQPGLVDAELVLHQLQCNGVLEGIRICRKGFPSRLIYSEFKQRYSILAPNAIPQG....F
3I5G_Myo2_D.pealeii KTPGLIDAALVLHQLRCNGVLEGIRICRKGFPNRIIYSEFKQRYSILAPNAVPSG....F
5N6A_Myo2_B.taurus KSPGVIDNPLVMHQLRCNGVLEGIRICRKGFPNRILYGDFRQRYRILNPAAIPEGQ...F
2Y8I_Myo2_D.discoideum QLPAKLEDKVVLDQLRCNAVLEGIRITRKGFPNRIIYADFVKRYYLLAPNVPRDA.....
1OE9_Myo5_G.gallus KFPFTFDEKRAVQQLRACGVLETIRISAAGFPSRWTYQEFFSRYRVLMKQKDVLS.....
4ZLK_Myo5_M.musculus KFPFTFDEKRAVQQLRACGVLETIRISAAGFPSRWTYQEFFSRYRVLMKQKDVLG.....
5KG8_Myo10_H.sapiens KMPDQFDQAVVLNQLRYSGMLETVRIRKAGYAVRRPFQDFYKRYKVLMRN...LAL...P
4ANJ_Myo6_S.scrofa MTSHHFEGAQILSQLQCSGMVSVLDLMQGGFPSRASFHELYNMYKKYMPDKLARLDP...
670 680 690 700 710 720
FgMyoI_1-736 GTTEAAVKQILKDTSIPKEEWQMGVTKAFIKAPETLFALEHMRDRYWHNMATRIQRMWRA
1LKX_Myo1_D.discoideum GTAKQATELILQQHNIDKEEIRMGKTKVFIRNPTTLFYFEEKRELEMP............
5V7X_Myo1_R.norvegicus GPARSGVEVLFNELEIPVEEYSFGRSKIFIRNPRTLFQLEDLRKQRLEDLATLIQKIYRG
1DFL_Myo2_A.irradians VDGKTVSEKILAGLQMDPAEYRLGTTKVFFKAG.VLGNLEEMRDERLSKIISMFQAHIRG
2EC6_Myo2_P.magellanicus VDGKTVSEKILTGLQMDPSEYRLGTTKVFFKAG.VLGNLEEMRDERLSKIISMFQAHIRG
3I5G_Myo2_D.pealeii ADGKVVTDKALSALQLDPNEYRLGNTKVFFKAG.VLGMLEDMRDERLSKIISMFQAHIRG
5N6A_Myo2_B.taurus IDSRKGAEKLLGSLDIDHNQYKFGHTKVFFKAG.LLGLLEEMRDERLSRIITRIQAQSRG
2Y8I_Myo2_D.discoideum EDSQKATDAVLKHLNIDPEQYRFGITKIFFRAG.QLARIEEARE................
1OE9_Myo5_G.gallus .DRKQTCKNVLEKLILDKDKYQFGKTKIFFRAG.QVAYLEKIRADKLRAACIRIQKTIRG
4ZLK_Myo5_M.musculus .DRKQTCKNVLEKLILDKDKYQFGKTKIFFRAG.QVAYLEKLRADKLRAACIRIQKTIRG
5KG8_Myo10_H.sapiens EDVRGKCTSLLQLYDASNSEWQLGKTKVFLRES.LEQKLEKRREEE..............
4ANJ_Myo6_S.scrofa ...RLFCKALFKALGLNEIDYKFGLTKVFFRPGKFAEFDQIMKSDPDHLAELVKRVNHWL

```
                          730
FgMyoI_1-736              YLAYRAES....................................................
1LKX_Myo1_D.discoideum    ............................................................
5V7X_Myo1_R.norvegicus    WKCRTHFLLMKGLNDIFEAQKAIEWHEDYKDDDDK.........................
1DFL_Myo2_A.irradians     YLIRKAYKKLQDQRIGLSVIQRNIRKWLVLRNWQWWKLYSKVKP................
2EC6_Myo2_P.magellanicus  YLIRKAYKKLQDQRIGLSVIQRNIRKWLVLRNWQWWKLYAKVKP................
3I5G_Myo2_D.pealeii       YLMRKAYKKLQDQRIGLTLIQRNVRKWLVLRNWEWWRLFNKVKPLL..............
5N6A_Myo2_B.taurus        VLSRMEFKKLLERRDSLLIIQWNIRAFMGVKNWP..........................
2Y8I_Myo2_D.discoideum    ............................................................
1OE9_Myo5_G.gallus        WLMRKKYMRMRRGDA.............................................
4ZLK_Myo5_M.musculus      WLLRKRYLCMQR................................................
5KG8_Myo10_H.sapiens      ............................................................
4ANJ_Myo6_S.scrofa        ICSRWKKVQWCSLSVIKLKNKIKYRAEAVSKGEELFTGVVPILVELDGDVNGHKFSVSGE

FgMyoI_1-736              ............................................................
1LKX_Myo1_D.discoideum    ............................................................
5V7X_Myo1_R.norvegicus    ............................................................
1DFL_Myo2_A.irradians     ............................................................
2EC6_Myo2_P.magellanicus  ............................................................
3I5G_Myo2_D.pealeii       ............................................................
5N6A_Myo2_B.taurus        ............................................................
2Y8I_Myo2_D.discoideum    ............................................................
1OE9_Myo5_G.gallus        ............................................................
4ZLK_Myo5_M.musculus      ............................................................
5KG8_Myo10_H.sapiens      ............................................................
4ANJ_Myo6_S.scrofa        GEGDATYGKLTLKFICTTGKLPVPWPTLVTTFGYGVQCFARYPDHMRQHDFFKSAMPEGY

FgMyoI_1-736              ............................................................
1LKX_Myo1_D.discoideum    ............................................................
5V7X_Myo1_R.norvegicus    ............................................................
1DFL_Myo2_A.irradians     ............................................................
2EC6_Myo2_P.magellanicus  ............................................................
3I5G_Myo2_D.pealeii       ............................................................
5N6A_Myo2_B.taurus        ............................................................
2Y8I_Myo2_D.discoideum    ............................................................
1OE9_Myo5_G.gallus        ............................................................
4ZLK_Myo5_M.musculus      ............................................................
5KG8_Myo10_H.sapiens      ............................................................
4ANJ_Myo6_S.scrofa        VQERTIFFKDDGNYKTRAEVKFEGDTLVNRIELKGIDFKEDGNILGHKLEYNYNSHNVYI

FgMyoI_1-736              ............................................................
1LKX_Myo1_D.discoideum    ............................................................
5V7X_Myo1_R.norvegicus    ............................................................
1DFL_Myo2_A.irradians     ............................................................
2EC6_Myo2_P.magellanicus  ............................................................
3I5G_Myo2_D.pealeii       ............................................................
5N6A_Myo2_B.taurus        ............................................................
2Y8I_Myo2_D.discoideum    ............................................................
1OE9_Myo5_G.gallus        ............................................................
4ZLK_Myo5_M.musculus      ............................................................
5KG8_Myo10_H.sapiens      ............................................................
4ANJ_Myo6_S.scrofa        MADKQKNGIKVNFKIRHNIEDGSVQLADHYQQNTPIGDGPVLLPDNHYLSYQSALSKDPN

FgMyoI_1-736              ..........................
1LKX_Myo1_D.discoideum    ..........................
5V7X_Myo1_R.norvegicus    ..........................
1DFL_Myo2_A.irradians     ..........................
2EC6_Myo2_P.magellanicus  ..........................
3I5G_Myo2_D.pealeii       ..........................
5N6A_Myo2_B.taurus        ..........................
2Y8I_Myo2_D.discoideum    ..........................
1OE9_Myo5_G.gallus        ..........................
4ZLK_Myo5_M.musculus      ..........................
5KG8_Myo10_H.sapiens      ..........................
4ANJ_Myo6_S.scrofa        EKRDHMVLLEFVTAAGITHGMDELYK
```
