# Supplementary material for: Structural basis of Fusarium myosin I inhibition by phenamacril
Source: PLoS Pathog. 2020 Mar 12;16(3):e1008323. doi: 10.1371/journal.ppat.1008323 (PMC7100991; doi:10.1371/journal.ppat.1008323)
Supplement: S2 Table — Pocket residues are labeled with variable pocket residues highlighted in green. F.: Fusarium; M.: Magnaporthe; B.: Botrytis; A.: Aspergillus; S.: Saccharomyces; H.: Homo; D.: Dictyostelium; *: Amino acid whose mutation cause high or medium resistance to phenamacril. (PDF) [file ppat.1008323.s009.pdf]

S2 Table. Sequence alignment of the myosin I motor domains of phenamacril-sensitive (red box) and phenamacril-resistant species.

Pocket residues are labeled with variable pocket residues highlighted in green. F. : *Fusarium*; M. : *Magnaporthe*; B. : *Botrytis*; A. : *Aspergillus*; S.: *Saccharomyces*; H. : *Homo;* D.: *Dictyostelium*; *: Amino acid whose mutation cause high or medium resistance to phenamacril.

```
                    1        10                    20        30        40
F.graminearum       MGISRRPKNKGAG..........AAADGASGGAKPKKATFETTKKKEIGVSDLTLLSKV
F.fujikuroi         MGISRRPKNKGAG..........AAADGASGGAKPKKATFETTKKKEIGVSDLTLLSKV
F.oxysporum         MGISRRPKNKGAG..........AAADGASGGAKPKKATFETTKKKEIGVSDLTLLSKV
F.verticillioides   MGISRRPKNKGAG..........AAADGASGGAKPKKATFETTKKKEIGVSDLTLLSKV
F.avenaceum         MGISRRPKNKGAG..........PAADGANGVAKPKKATFETTKKKEIGVSDLTLLSKV
F.solani            MGISRRPKNKDAR..........AGAEAG...AKPKKATFETTKKKEIGVSDLTLLSKV
M.grisea            MGITRRGKDKAAAGQ........AVAGGASGGRARPKKATFETSKKKDVGVSDLTLLSKV
B.cinerea           MGISRRPKGNNNAS.........AAESAPSGKPNIQKAQFDTTKKKEVGVSDLTLISKV
A.flavus            MGHSRRPVGGEKKSRGFGRSKVADVGDGRQAGKPQVKKATFETTKKKDIGVSDLTLLSKI
S.cerevisiae        MAILKRGARKKVHQEP............AKRSANIKKATFDSSKKKEVGVSDLTLLSKI
H.sapiens           ............................................................
D.discoideum        ............................................................
```

```
                   50        60        70        80        90       100
F.graminearum       SNEAINENLKKRFEGREIYTYIGHVLVSVNPFRDLGIYTDDVLQSYMGKNRLEMPPHVFA
F.fujikuroi         SNEAINDNLKKRFEGHEIYTYIGHVLVSVNPFRDLGIYTDQVLESYMGKNRLEMPPHVFA
F.oxysporum         SNEAINENLKKRFEGHEIYTYIGHVLVSVNPFRDLGIYTDQVLESYMGKNRLEMPPHVFA
F.verticillioides   SNEAINENLKKRFEGREIYTYIGHVLVSVNPFRDLGIYTDQVLESYMGKNRLEMPPHVFA
F.avenaceum         SNEAINENLKKRFEGAEIYTYIGHVLVSVNPFRDLGIYTDQVLESYMGKNRLEMPPHVFA
F.solani            SNEAINENLKKRFEGAEIYTYIGHVLVSVNPFRDLGIYTDQVLDTYKGKNRLEMPPHVFA
M.grisea            SNEAINENLQKRFEGREIYTYIGHVLVSVNPFRDLGIYTDQVLDSYKGKNRLEMPPHVFA
B.cinerea           SNEAINENLKKRFDNREIYTYIGHVLVSVNPFRDLGIYTDAVLDSYKGKNRLEMPPHVFA
A.flavus            SNEAINDNLKLRFEHDEIYTYIGHVLVSVNPFRDLGIYTDNVLDSYRGKNRLEVPPHVFA
S.cerevisiae        SDEAINENLKKRFLNATIYTYIGHVLISVNPFRDLGIYTDAVMNKYKGKNRLEVPPHVFA
H.sapiens           ..............................PFKQMPYFGEKEIEMYQGAAQYENPPHIYA
D.discoideum        .......................................DIKAYNGRYKYEMPPHIYA
```

```
                  110       120       130       140       150       160
F.graminearum       IAEASYYNMKAYSDNQCVIISGESGAGKTEAAKRIMQYIASVSGGESGDIKQIKDMVLAT
F.fujikuroi         IAEASYYNMKAYSDNQCVIISGESGAGKTEAAKRIMQYIASVSGGESGDIKQIKDMVLAT
F.oxysporum         IAEAAYYNMKAYSDNQCVIISGESGAGKTEAAKRIMQYIASASGGESGDIKQIKDMVLAT
F.verticillioides   IAEASYYNMKAYSDNQCVIISGESGAGKTEAAKRIMQYIASVSGGESGDIKQIKDMVLAT
F.avenaceum         IAEASYYNMKAYSDNQCVIISGESGAGKTEAAKRIMQYIASVSGGESGDIKQIKDMVLAT
F.solani            IAESAYYNMKAYSDNQCVIISGESGAGKTEAAKRIMQYIASVSGGESGDIKQIKDMVLAT
M.grisea            IAESAYYNMKAYKDNQCVIISGESGAGKTEAAKRIMQYIASVSGGDSTDIQQIKDMVLAT
B.cinerea           VAESAYYNMNGYKDNQCVIISGESGAGKTEAAKRIMQYIANVSGGSNSSIQEIKDMVLAT
A.flavus            VAESSYYNMKSYKDNQCVIISGESGAGKTEAAKRIMQYIASVSGGSDSSIQQTKDMVLAT
S.cerevisiae        IAESMYYNMKSYNENQCVIISGESGAGKTEAAKRIMQYIAAASSTHTESIGKIKDMVLAT
H.sapiens           LADNMYRNMIIDRENQCVIISGESGAGKTVAAKYIMSYISRVSGG.GTKVQHVKDIILQS
D.discoideum        LANDAYRSMRQSQENQCVIISGESGAGKTEASKKIMQFLTFVSSNQSPNGERISKMLLDS
```

```
                                                                *S217
                                                              *K216
                                                            E215
                                                          L214
                                                        L213
                  170       180       190       200       210       220
F.graminearum       NPLLESFGNAKTLRNNNSSRFGKYLQIYFNTQGEPVGADITNYLLEKSRVVGQITNERNF
F.fujikuroi         NPLLESFGNAKTLRNNNSSRFGKYLQIYFNAQGEPVGADITNYLLEKSRVVGQITNERNF
F.oxysporum         NPLLESFGNAKTLRNNNSSRFGKYLQIYFNAQGEPVGADITNYLLEKSRVVGQITNERNF
F.verticillioides   NPLLESFGNAKTLRNNNSSRFGKYLQIYFNAQGEPVGADITNYLLEKSRVVGQITNERNF
F.avenaceum         NPLLESFGNAKTLRNNNSSRFGKYLQIYFNTQGEPVGADITNYLLEQSRVVGQITNERNF
F.solani            NPLLESFGNAKTLRNNNSSRFGKYLQIYFNAQGEPVGADITNYLLEKTRVVGQITNERNF
M.grisea            NPLLESFGNAKTLRNNNSSRFGKYLQIHFNSVGEPVGADITNYLLEKSRVVGQITNERNF
B.cinerea           NPLLESFGNAKTLRNNNSSRFGKYLQLQFNAQGEPVGADITNYLLEKTRVVTQIKDERNF
A.flavus            NPLLESFGNAKTLRNNNSSRFGKYLELEFNANGEPVGANITNYLLEKSRVVGQIANERNF
S.cerevisiae        NPLLESFGCAKTLRNNNSSRHGKYLEIKFNNQFEPCAGNITNYLLEKQRVVSQIKNERNF
H.sapiens           NPLLEAFGNAKTVRNNNSSRFGKYFEIQFSPGGEPDGGKISNFLLEKSRVVMRNPGERSF
D.discoideum        NPLLEAFGNAKTLRNDNSSRFGKYMEMQFNAVGSPIGGKITNYLLEKSRVVGRTQGERSF
```

```
                  230       240       250       260       270       280
F.graminearum       HIFYQFAKGASQQYRETFGVQK.PETYVYTSRSKCLDVDGIDDLAEFEDTLNAMKVIGLS
F.fujikuroi         HIFYQFAKGASQQYRETFGVQK.PETYVYTSRSKCLDVDGIDDLAEFQDTLNAMKVIGLT
F.oxysporum         HIFYQFAKGASQQYRETFGVQK.PETYVYTSRSKCLDVDGIDDLAEFQDTLNAMKVIGLT
F.verticillioides   HIFYQFAKGASQQYRETFGVQK.PETYVYTSRSKCLDVDGIDDLAEFQDTLNAMKVIGLT
F.avenaceum         HIFYQFAKGASEQYRQTFGVQK.PETYVYTSRSKCLDVDGIDDLAEFEDTLNAMKVIGLS
F.solani            HIFYQFTKGASQQYRETFGIQK.PETYVYTSRSKCFDVDGIDDLAEFQDTLNAMKIIGLS
M.grisea            HIFYQFTKGASEHYRQMFGIQK.PETYIYTSRSKCLDVDGIDDLAEFQDTLNAMKVIGLS
B.cinerea           HIFYQFTKGASQAYRENFGIQQ.PSQYLYTSKAGCFDVDGIDDLAEYQDTLNAMKIIGLS
A.flavus            HIFYQFTKAAPQKYRDMFGVQQ.PQSYLYTSRSKCFDVPGVDDNAEFRDTLNAMGVIGMS
S.cerevisiae        HIFYQFTKGASDAYRQTFGVQK.PEQYVYTAAAGCISAETIDDLQDYQETLKAMRVIGLG
H.sapiens           HIFYQLIEGASAEQKHSLGITS.MDYYYYLSLSGSYKVDDIDDRREFQETLHAMNVIGIF
D.discoideum        HIFYQMLKGLSQSKLNELGLTPNAPAYEYLKKSGCFDVSTIDDSGEFKIIVKAMETLGLK
```

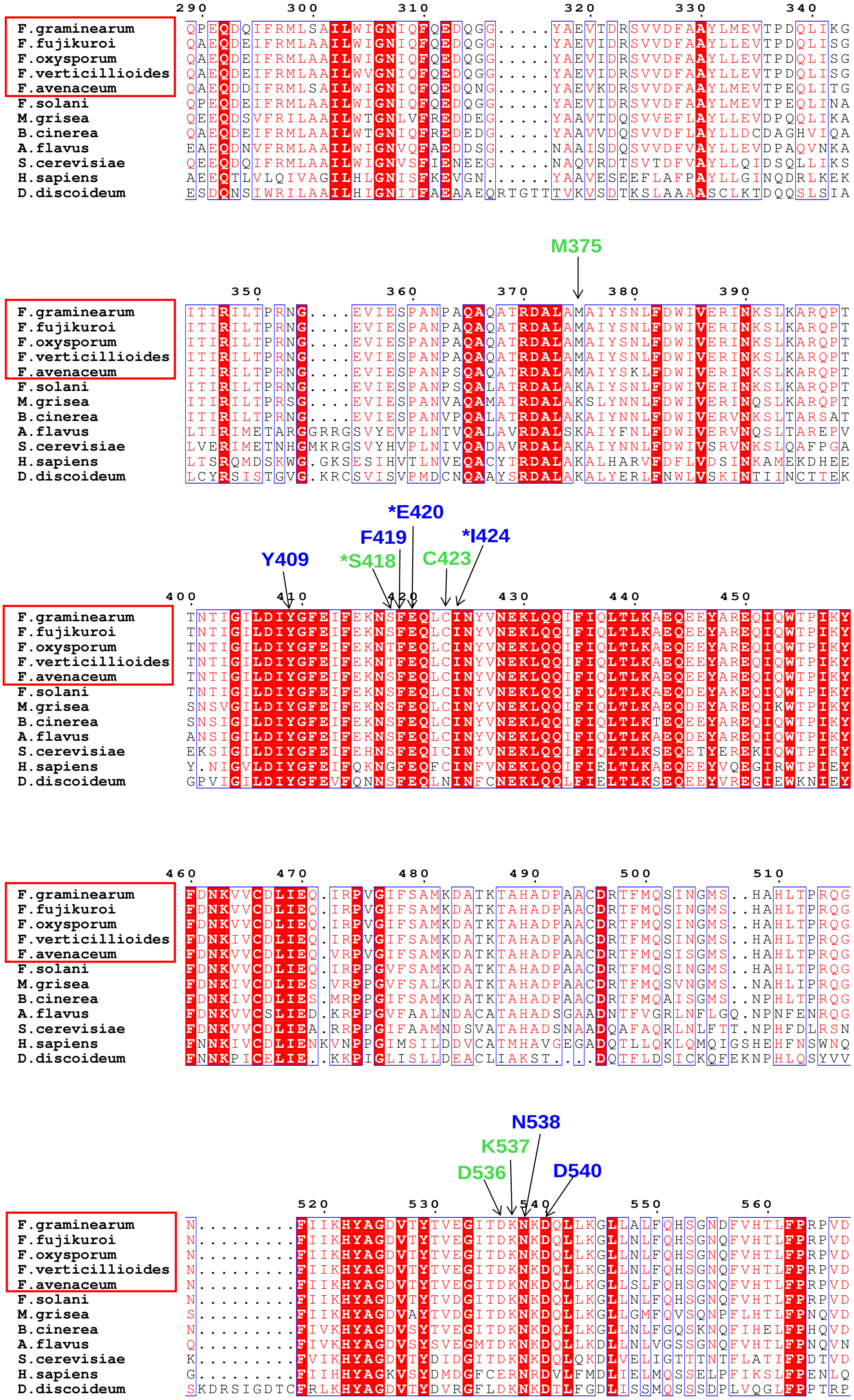
F.graminearum
F.fujikuroi
F.oxysporum
F.verticillioides
F.avenaceum
F.solani
M.grisea
B.cinerea
A.flavus
S.cerevisiae
H.sapiens
D.discoideum
M375
Y409
F419
*E420
*S418
C423
*I424
N538
K537
D536
D540

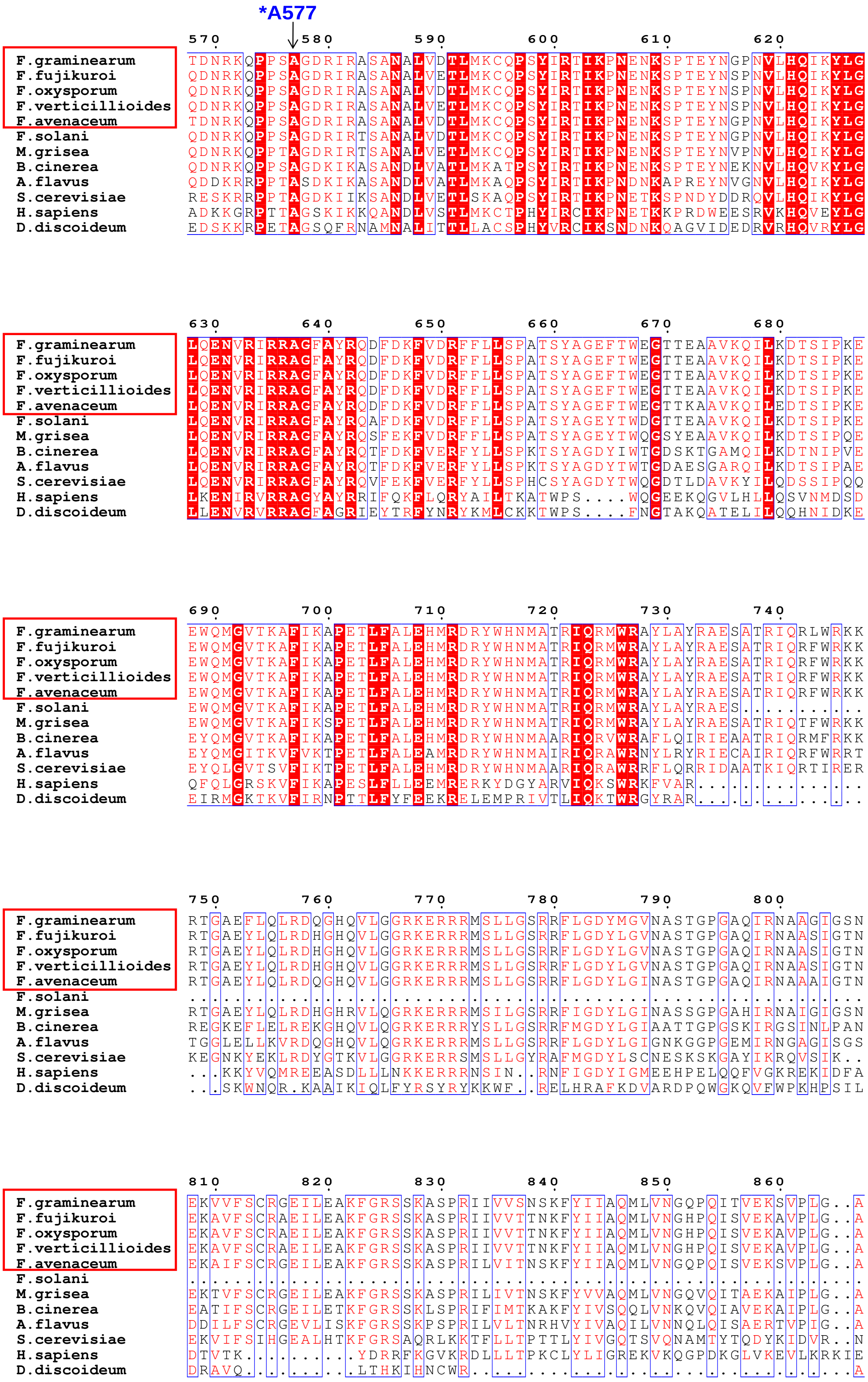
*A577
570
580
590
600
610
620
630
640
650
660
670
680
690
700
710
720
730
740
750
760
770
780
790
800
810
820
830
840
850
860
F.graminearum
F.fujikuroi
F.oxysporum
F.verticillioides
F.avenaceum
F.solani
M.grisea
B.cinerea
A.flavus
S.cerevisiae
H.sapiens
D.discoideum

```
                     870       880       890       900       910       920
F.graminearum      IKFIGVSSARDDWFSLGIGSPQEADPLMNCIFKTEMFTQMQRVMPGG.FNLKIAETIEYA
F.fujikuroi        IKFIGASSARDDWFSLGIGSPQEPDPLMNCMLKTEMFTQMQRVMPGG.FNLKIAETIEYA
F.oxysporum        IKFIGASSARDDWFSLGIGSPQEPDPLMNCMLKTEMFTQMQRVMPGG.FNLKIAETIEYA
F.verticillioides  IKFIGASSARDDWFSLGIGSPQEPDPLMNCMFKTEMFTQMQRVMPGG.FNLKIAETIEYA
F.avenaceum        IKFVGASSARDDWFSLGIGSPQEADPLMNCMFKTEMFTQMQRVMPGG.FNLKIAETIEYA
F.solani           ............................................................
M.grisea           IKFIGASSSRDDWFSLGVGSPQEADPLLNCVLKTEMFTQMERVMPGG.FNLKIGDSIEYA
B.cinerea          IKFVSISTCRDDWFSLGVGSPQEADPLLTCVFKTELFTHMQGAMPGGGFNLKIGDSIEYA
A.flavus           IKAVSTSNLKDDWFSLIIGG.QEPDPLINCVFKTEFFTHLQTALRGQ.LNLKVSENIEYN
S.cerevisiae       IQAVSLTNLQDDWVAIKLASSGQPDPLINTYFKTELITHLK..RLNDKIQIKIGSAIEYQ
H.sapiens          IERILSVSLSTMQDDIFILHEQEYDSLLESVFKTEFLSLLAKRYEEKTQKQLPLKFSNTL
D.discoideum       EKMILSLGAGQNHMRQKVMAYDIFHGKKKWDFRRHFDADYLEKPSNPNQQKYVLAMQNLF
```

```
                      930       940       950       960       970       980
F.graminearum      KKPGKLQQVKVLKD..SQVPADYYKSGAVHCQPGEPPSSVSKPTPKGKPVPPRPITRGKL
F.fujikuroi        KKPGKMQQVKVLKD..SQLPVDYYKSGAVHTQPGEPPSSISRPTPKGKPVPPRPITRGKL
F.oxysporum        KKPGKMQQVKVLKD..SQLPVDYYKSGAVHTQPGEPPSSVSRPTPKGKPVPPRPITRGKL
F.verticillioides  KKPGKMQQVKVLKD..SQLPVDYYKSGAVHTQPGEPPSSISRPTPKGKPVPPRPITRGKL
F.avenaceum        KKPNKMQQVKVMKD..SQLPVDYYKSGAVHTQPGEPPNSGSKPTPKGKPVPPRPITRGKL
F.solani           ............................................................
M.grisea           KKPGKMQVVKVLKD..SPNPVDFYKSGAVHTQQGEPPNSVSRPTPKGKPVPPRPITRGKL
B.cinerea          KKPNKMQLIKVVKD..SQQAQDHYKSATIHTQAGEPPTSRSKPLPKGKPVAPKPFTSGRL
A.flavus           KKPGKLATVKAIKDPAASPNVDTYKSHTIHTSPGEPPSSVSKPTPKAKQVAARPVTKGKL
S.cerevisiae       KKPGKLHSVKCQINESAPKYGDIYKSSTISVRRGNPPNSQVHKKPRKKSS....ISSGYH
H.sapiens          ELKLKKENWGPWSAGGSRQVQFHQGFGDLAVLKPSNKVLQVSIGPG.............L
D.discoideum       STYG..............................DTEVLFADYVIK.............V
```

```
                        990      1000      1010      1020      1030
F.graminearum      IKPGGPNGRPSRIQGNRAAKPRPGGGARAVPQPPVAVSAAASIPAAVP............
F.fujikuroi        IKPGGPNGRPSRIQGNRTAKPRPG.AGRAVPQPPAAVSNIAAVPAAAAQSTPRIAPRPGA
F.oxysporum        IKPGGPNGRPSRIQGNRTAKPRPG.AGRAVPQPPAAVSNIAAVPAAATQSTPRIAPRPGA
F.verticillioides  IKPGGPNGRPSRIQGNRTAKPRPG.AGRAVPQPPAAVSNIAAVPAAATQSTSRIAPRPGA
F.avenaceum        IRPGGPNGRPSRIQAT...KPRPGAGTRAVPQPPAALAAAASIPAAATQATPRIAPRPVA
F.solani           ............................................................
M.grisea           IRPGGPNGRPARGTTNRTPQPRPGGASASAVASRPVPQAQP...................
B.cinerea          IKPGGPGGRPSKLTNGNRPTPKPVPSPAAARPVPTPAAAMPIPAPIPAAN..........
A.flavus           LRPGGPGGGPSKLASRPASRPTPKPQPLPQSQPATAQPIPAPQPAAVP............
S.cerevisiae       ASSSQATRRPVSIAAAQHVPTAPASRHSKKPAPPPPGMQNKAATRRSVPNPASTLTASKS
H.sapiens          PKNSRPTRRNTTQNTGYSSGTQNANYPVRAAPPPPG........................
D.discoideum       NPKGVPQRRGIVVTGTNIYKHDPKNYKVKKWGTPLV........................
```

```
                            1040      1050      1060      1070      1080
F.graminearum      ...APAAATHNALPSHAKAASAAGRAPPPPPPPAAPARPPSPPRVMAKVLYDFAGQRENE
F.fujikuroi        NAATPIPAAHNALPSHTRNASGAGRAPPPPPP.AVAARPPSPPKVMAKVLYDFAGQRENE
F.oxysporum        NAAAPIPAAHNALPSHTRNASGAGRAPPPPPP.PVAARPPSPPKVMAKVLYDFAGQRENE
F.verticillioides  NAAAPVPAAHNALPSHTRNASGAGRAPPPPPPPAAAARPPSPPKVMAKVLYDFAGQRENE
F.avenaceum        ASAAPTPAAHNPLPSHTRNASGAGRAPPPPPPPAAPARPPSPPKVLAKVLYDFAGQRENE
F.solani           ............................................................
M.grisea           ...QAQAQVAASIPVRTQQQSQTSSASVRAPPPPPPAAPPAKAKIMAKVLYDFAGQKENE
B.cinerea          .PIAASIPVHTRNPSLQSAQSTRAVPPPPPPAPPAPPPAPASKEPQYRVLYEFAGQSANE
A.flavus           .....RPVPQPVAAAAASHTRNASSGSVRAPPPPPPASPPAPKKATAKALYDFTSAQSNE
S.cerevisiae       NARPSPPTAATRATPAAAAMGSDRQANIPPPPPPPPPSSKPKEPMFEAAYDFSGSGSPSE
H.sapiens          ...........YHQNGVIRNQYVPYPHAPGSQRSNQKSLYTSMARPPLPRQQSTSSDRVS
D.discoideum       ...........DVTSISIS...........PMADTFLVLHCKAPQRDFVLDLGCNGYEAV
```

```
                  1090      1100      1110      1120      1130              1140
F.graminearum      LSIAAGEIVEIVQKESNGWWLAKNPQTAQQAWVPAAYVEEQAPPAP.......RAPPAPP
F.fujikuroi        LTITANEIVEIVQKESNGWWLAKNPQTAQQAWVPAAYVEEQAPPAP.......RAPPAPP
F.oxysporum        LTITANEIVEIVQKESNGWWLAKNPQTAQQAWVPAAYVEEQAPPAP.......RAPPAPP
F.verticillioides  LTITANEIVEIVQKESNGWWLAKNPQTAQQAWVPAAYVEEQAPPAP.......RAPPAPP
F.avenaceum        LTITAGQLVEIVQKETNGWWLAKNPNTEQQAWVPAAYVEEQAPPAP.......RAPPAPP
F.solani           ............................................................
M.grisea           MSIKEGDLIEIVQKENNGWWLAKS..GNQQAWVPAAYVEEQKQAPPPVAASRPPPPAPPA
B.cinerea          FSLKQGEIVTVLQKETNGWWLTKN..VRGQGWAPTAYLEEVAPPPPPPIASRPTPPPPPG
A.flavus           LDIRAGDVVQIVSKEGNGWWLCMNMATSVQGWTPQAYLEEQVAPTPKPAPPPPPPAAPRA
S.cerevisiae       LPLKKGDVIYITREEPSGWSLGKLLDGSKEGWVPTAYMKPHSGNNN..............
H.sapiens          QTPESLDFLKVPDQGAAGVRRQTTSRPPPAGGRPKPQPKPKPQVPQ..............
D.discoideum       SEITTVIVQQVLKLTGVKLSVQFTSSITYNNARPKGS.......................
```

```
                                                 1150      1160      1170
F.graminearum       ................RSKPTPPAPPAKRPAAG...RKPAELQQ.RDSGMSLNT.PNGSD
F.fujikuroi         ................RSKPTPPAPPAKRPAAN...RKPAELQQ.RDSGMSLNT.PNGGD
F.oxysporum         ................RSKPTPPAPPAKRPAAN...RKPAELQQ.RDSGMSLNT.PNGGD
F.verticillioides   ................RSKPTPPAPPAKRPAAN...RKPAELQQ.RDSGMSLNT.PNGGD
F.avenaceum         ................RSKPMPPAPPAKRPAAG...RKPAELQQ.RDSGMSLNT.PNGGS
F.solani            ............................................................
M.grisea            ................ANGKNKPLPPAKRPAAG...KKPASLQP.RDSGMSLNG.SDG..
B.cinerea           PPKMNGA....NGAAVRSKPTPPAPPAKRPVAG...RKPAPPPAPRDSGMSISSNGSGNN
A.flavus            SPVPSANGAAATAAAAKAKPAPPAPPAKRPNMAGRKAVPAPPPAPRDSAVSMNSHDSSGG
S.cerevisiae        ......................................IPTPPQNRDVPKPVLNSVQHDN
H.sapiens           ....................................CKALYAYDAQDTDELSFNANDIID
D.discoideum        .....................................................DTILTFA

                    1180      1190      1200           1210
F.graminearum       SRSSTP....TPSLGGSLADALLARKNAMQ........KEKEDDDDW
F.fujikuroi         SRSSTP....TPSLGGSLADALLARKNAMQ........KEKEDDDDW
F.oxysporum         SRSSTP....TPSLGGSLADALLARKNAMQ........KEKEDDDDW
F.verticillioides   SRSSTP....TPSLGGSLADALLARKNAMQ........KEKEDDDDW
F.avenaceum         SRSSTP....TPSLSGSLADALLARKNAMQ........KEKDDDDDW
F.solani            ...............................................
M.grisea            SRSNTP....TPSLGNSLADALLARKQAMA........KKDDDDDW.
B.cinerea           SGRSTP....TPSLAGGLAEALRARQSAMQGN......AKKEDEDDW
A.flavus            SGRGTPNSTSNASLAGGLAEALRARQHAMQG........KHDDDDEW
S.cerevisiae        TSANVIPAAAQASLGDGLANALAARANKMRLESDDEEANEDEEEDDW
H.sapiens           IIKEDPSGWWTGRLRGKQGLFPNNYVTKI..................
D.discoideum        PINNDPKLIGSQFKKGKGNQATIQFKD....................
```
